# Fruits for Prevention and Treatment of Cardiovascular Diseases

**DOI:** 10.3390/nu9060598

**Published:** 2017-06-13

**Authors:** Cai-Ning Zhao, Xiao Meng, Ya Li, Sha Li, Qing Liu, Guo-Yi Tang, Hua-Bin Li

**Affiliations:** 1 Guangdong Provincial Key Laboratory of Food, Nutrition and Health, Department of Nutrition, School of Public Health, Sun Yat-sen University, Guangzhou 510080, China; zhaocn@mail2.sysu.edu.cn (C.-N.Z.); mengx7@mail2.sysu.edu.cn (X.M.); liya28@mail2.sysu.edu.cn (Y.L.); liuq248@mail2.sysu.edu.cn (Q.L.); tanggy5@mail2.sysu.edu.cn (G.-Y.T.); 2 School of Chinese Medicine, Li Ka Shing Faculty of Medicine, The University of Hong Kong, Hong Kong 999077, China; 3 South China Sea Bioresource Exploitation and Utilization Collaborative Innovation Center, Sun Yat-sen University, Guangzhou 510006, China

**Keywords:** fruit, cardiovascular disease, coronary heart disease, stroke, hypertension, mechanisms of action

## Abstract

Cardiovascular diseases (CVDs) are leading global health problems. Accumulating epidemiological studies have indicated that consuming fruits was inversely related to the risk of CVDs. Moreover, substantial experimental studies have supported the protective role of fruits against CVDs, and several fruits (grape, blueberry, pomegranate, apple, hawthorn, and avocado) have been widely studied and have shown potent cardiovascular protective action. Fruits can prevent CVDs or facilitate the restoration of morphology and functions of heart and vessels after injury. The involved mechanisms included protecting vascular endothelial function, regulating lipids metabolism, modulating blood pressure, inhibiting platelets function, alleviating ischemia/reperfusion injury, suppressing thrombosis, reducing oxidative stress, and attenuating inflammation. The present review summarizes recent discoveries about the effects of fruits on CVDs and discusses potential mechanisms of actions based on evidence from epidemiological, experimental, and clinical studies.

## 1. Introduction

Cardiovascular diseases (CVDs) are defined as disorders of the heart and vessels, and include coronary heart disease (CHD) and stroke. According to the WHO report, CVDs are responsible for 17.5 million deaths in 2012 (7.4 and 6.7 million due to CHD and stroke, respectively), accounting for 31% of all global deaths a year, constituting the leading causes of death worldwide [1]. Thus, studies on CVDs have drawn great attention around the world.

Diet represents the most important modifiable factor to prevent CVDs. There is evidence that plant-based dietary patterns are associated with lower risk of CVDs [2]. Among the most important key components, fruit has been suggested to play a major role in preventing CVDs [3]. Several epidemiological studies demonstrated that fruit intake was inversely associated with the risk of cardiovascular events [4,5,6,7]. It is estimated that a diet low in fruits is the third most important risk factor of CVDs following high blood pressure (BP) and cigarette smoking, accounting for more than 5 million deaths worldwide in 2010 [8]. In addition, much experimental evidence supports the protective role of fruit against CVDs. Furthermore, several fruits, such as grape, blueberry, pomegranate, apple, hawthorn, and avocado, have been widely studied and have shown strong cardiovascular protective effects. Additionally, the effectiveness of fruit intake in the primary prevention of CVDs has been revealed by growing clinical data in patients with high metabolic risk factors (hypertension, dyslipidemia, diabetes, and overweight/obesity). Currently, the common method of controlling CVDs is the use of long-term pharmacotherapy. Nevertheless, drugs are not effective on all patients and have side effects that may aggravate the patients’ symptoms and signs. Some fruits (extract) possess similar or even more potent anti-hypertensive, lipid-lowering, and hypoglycemic activities, which has inspired many researchers to explore new therapies for CVDs [9,10,11,12]. The cardioprotective mechanisms of fruits are not entirely clear, but their outstanding antioxidant and free radical scavenging properties are considered principal [13]. In this context, people have paid more attention to natural products rich in polyphenols, a group of compounds characterized by the presence of an aromatic ring and phenolic hydroxyl groups [14,15,16,17,18,19,20,21,22,23,24,25]. Polyphenols are obtained through daily diets because they cannot be synthesized or stored in the human body, and fruit is one of the main dietary sources of polyphenols [26]. The richest sources of fruit polyphenols are dark berries, such as grapes and blueberries [27]. Further, pomegranate, apple, hawthorn, and avocado are also frequently consumed polyphenols-rich fruits. There is evidence that fruit rich in polyphenols helps to control CVDs.

This review aims to summarize the effects of fruit on CVDs based on evidence from epidemiological, experimental, and clinical studies, and special attention is paid to the mechanisms of action.

## 2. Epidemiological Studies

Epidemiological evidence supports that diets rich in fruit delay the onset, and attenuate the severity, of CVDs (Table 1).

Evidence from the China Kadoorie Biobank Study showed that consuming fresh fruits daily decreased systolic blood pressure (SBP) by 4.0 mmHg and blood glucose level by 0.5 mmol/L, which was inversely associated with the risks of cardiovascular death (hazard ratios (HRs): 0.60, 95% confidence intervals (CIs): 0.54–0.67) and major CVDs, i.e., CHD (HR: 0.66, 95% CI: 0.58–0.75), ischemic stroke (HR: 0.75, 95% CI: 0.72–0.79), and hemorrhagic stroke (HR: 0.64, 95% CI: 0.56–0.74), as compared with participants who never or rarely consumed fresh fruit [4]. In addition, two cohort studies of women aged 35–69 [5] and over 70 years [6], respectively, showed that consuming fruits reduced the risk of total CVD mortality. Additionally, a Japanese study suggested that frequent intake of citrus fruit protected against CVDs; the HR for near-daily intake versus infrequent intake of citrus fruit was 0.57 (95% CI: 0.33–1.01) in men and 0.51 (95% CI: 0.29–0.88) in women [7].

A cohort study of women in Shanghai showed a protective role of higher dietary total fruit and vegetable intake in CHD. Moreover, the study suggested that this association was primarily driven by fruit. The corresponding HRs for fruit and vegetable intake were 0.62 (95% CI: 0.37–1.03) and 0.94 (95% CI: 0.59–1.50), respectively [28]. In addition, a meta-analysis of 23 prospective cohort studies of 937,665 participants and 18,047 CHD patients showed that fruit consumption was inversely associated with a risk of CHD. Compared with those who consumed the lowest total fruits, the relative risk (RR) of CHD was 0.86 (95% CI: 0.82–0.91) for those consuming the highest, and the dose-response analysis indicated that the RR of CHD was 0.84 (95% CI: 0.75–0.93) per 300 g/day of total fruit intake [29]. For individual fruit, apple intake reduced the risk of acute coronary syndrome (ACS) by 3%, and the dose-response analysis indicated that the HR of ACS was 0.97 (95% CI: 0.93–1.01) per 25 g/day of apple intake [30].

In terms of stroke, cohorts of Swedish women and men suggested that consuming 3.1 servings/day of total fruits alleviated total stroke risk by 13% compared with 0.4 servings/day (95% CI: 0.78–0.97) [31]. Furthermore, the study also indicated that, among individual fruits, consumption of apple/pear particularly decreased the risk of total stroke (HR: 0.89, 95% CI: 0.80–0.98) [31], which was consistent with the result of a study in Netherlands of 20,069 adults [32]. The study in Netherlands also reported that consuming >120 g/day raw fruit decreased the risk of hemorrhagic stroke by 47% (95% CI: 0.28–1.01) compared with consuming ≤120 g/day raw fruits [33]. In addition, the Nurses’ Health Study showed that high citrus fruit/juice intake was related to a reduced risk of ischemic stroke (RR: 0.90, 95% CI: 0.77–1.05) [34]. Additionally, a cohort study with 20,024 participants recruited also proved that citrus fruits/juice intake was inversely associated with risk of ischemic stroke (HR: 0.69, 95% CI: 0.53–0.91) [35].

Epidemiological studies have suggested that fruit consumption was related to a reduction in cardiovascular risk factors. Hypertension is an independent risk factor of CHD and total stroke [36]. Three cohort studies all reported that higher fruit intake was correlated with the decreased risk of hypertension [37,38,39]. A study of US women showed that total fruit and vegetables consumption attenuated the risk of hypertension. In addition, after adjusting for lifestyle factors and other food intake, total fruit (*p* = 0.0004) but not total vegetables (*p* = 0.56) remained significantly and inversely correlated with risk of hypertension [37]. In addition, a study on residents from Ohasama, Japan, revealed an association between fruit and vegetable intake and the risk of hypertension. In the sex- and BMI-adjusted analysis, the highest quartile of fruit intake was associated with a significantly lower risk of hypertension (HR: 0.40, 95% CI: 0.21–0.74), whereas no association was observed for vegetable intake [38]. Moreover, a study consisting of three large longitudinal cohorts, Nurses’ Health Study, Nurses’ Health Study II, and Health Professionals Follow-up Study, suggested that long-term and increased consumption of whole fruits reduced the risk of hypertension [39]. Additionally, a case–control study in Korea was also in line with this view [40]. Furthermore, cross-sectional studies of patients with type 2 diabetes demonstrated that higher fruit intake was correlated with a lower burden of CVDs by decreasing carotid intima-media thickness (IMT), the prevalence of carotid plaque [41] and high-sensitive C-reactive protein (hs-CRP) levels [42], which have been well-established predictors for cardiovascular incidents.

Epidemiological studies indicated that dietary intake of polyphenols was associated with a low incidence of CVDs. The Nurses’ Health Study with 69,622 women involved showed that the RR for the fifth quintile of flavanone intake versus the lowest quintile was 0.81 (95% CI: 0.66–0.99) [34]. In addition, the relationship between flavonoids intake and CVDs in men was studied in the Health Professionals Follow-Up Study. The results revealed that higher anthocyanin intake was related with lower non-fatal myocardial infarction (MI) risk (HR: 0.87, 95% CI: 0.75–1.00), and higher flavanone intake was associated with decreased ischemic stroke risk (HR: 0.78, 95% CI: 0.62–0.97). The study also reported that over 90% dietary anthocyanins and flavanones came from fruits [43]. In addition, the association between flavonoid intake and ischemic stroke was evaluated in a cohort study of 20,024 participants. The study suggested that flavanone intake was inversely associated with a risk of ischemic stroke (HR: 0.72, 95% Cl: 0.55–0.95) [35]. Furthermore, the cardiovascular benefits of flavonoid and stilbene were estimated in a cross-sectional study of 1393 Chinese adults. The study showed that fruits including apple, plum, pear, and peach were the richest sources of flavonoids and stilbenes. Higher anthocyanin intake was related with elevated serum HDL-C (*p* = 0.001), and total flavonoid and flavonol intake was inversely associated with serum TG (*p* = 0.020, *p* = 0.035) and TG/HDL-C ratios (*p* = 0.040, *p* = 0.045) in female subjects. However, significant relationships were not found in male subjects [44].

However, a cohort of Italian women indicated no significant association between fruit intake and the risk of CHD after adjusting for the consumption of vegetables [45]. In addition, a cohort of men aged 50–59 years in France and Northern Ireland reported that there was no significant association between fruit intake and ACS [46]. A large scale cohort of five ethnic groups, i.e., African American, Native Hawaiian, Japanese American, Latino, and Caucasian showed that the consumption of fruits did not protect against ischemic heart disease, and the results did not vary among ethnic groups [47,48]. Additionally, a cohort of Swedish women (aged 49–83 years) suggested that the highest quintile of fruit intake did not significantly decrease the risk of heart failure compared with the lowest [49]. Results are inconsistent maybe because data regarding fruit intake in these cohort studies were obtained on the basis of dietary recall. The actual consumption of fruits can only be rudely assessed, partly because the number of items and the information about portion size were limited.

## 3. Experimental Studies

There has been accumulating evidence in vivo and in vitro supporting the cardiovascular protective properties of fruits and investigating the underlying mechanisms (Table 2). Six fruits are discussed in detail below because they have been widely studied and have shown potent cardiovascular protective effects, while the fruits that were less investigated are discussed in the section entitled “Other Fruits.”

### 3.1. Grape

Grapes are one of the most common and important fruits worldwide, and they are often consumed raw or after being converted to juice, wine, or jam.

#### 3.1.1. Protecting Endothelial Function

In CVDs, endothelial dysfunction is a systemic pathology of the endothelium, is caused by an imbalance between vasodilator and vasoconstrictor substances produced by (or acting on) the endothelium, and presents as impaired vascular endothelium-dependent relaxation and compliance, which is the primary change in early hypertension [50]. Growing experimental and clinical data highlight the importance of oxidative stress on endothelial dysfunction. Grape plays an essential role in repairing endothelial impairment for its potent antioxidant and free radical scavenging capacities. In a study, vascular benefits of whole grape powder were studied using the spontaneously hypertensive rat (SHR). The results showed that grape treatment elicited a reduction in BP, improved arterial relaxation, and increased vascular compliance [51]. Moreover, the relationship between endothelial protective function of grape seed proanthocyanidin extracts (GSPEs) and oxidative stress was studied in SHR and deoxycorticosterone acetate (DOCA)-salt hypertensive mice. The study indicated that GSPEs reduced endothelin (ET)-1 production but increased nitric oxide (NO) production, which exhibited improved endothelial function. Moreover, GSPEs ameliorated oxidative stress by improving superoxide dismutase (SOD) and catalase (CAT) activities and reducing malondialdehyde (MDA) formation [52,53]. Similarly, enzymatic extract of grape pomace (GP-EE) also induced endothelium- and NO^−^-dependent vasodilatation of both rat aorta and small mesenteric artery (SMA) segments, prevented contraction elicited by ET-1, and reduced superoxide anion radical (O_2_^−^) production [54]. Furthermore, another study showed that polyphenols in red grape skin and seeds increased endothelial progenitor cells viability, adhesion and migration, and prevented endothelial dysfunction by reducing reactive oxygen species (ROS) production [55]. In addition, red grape components increased the expression of endothelial nitric oxide synthase (eNOS) [56]. In vitro, human umbilical vein endothelial cells (HUVECs) were incubated with GSPEs to explore the signaling pathways of eNOS expression. The result suggested that the increased eNOS expression was attributed to the activation of 5′-AMP activated protein kinase (AMPK) and the increase in sirtuin-1 (SIRT-1) protein level, which was critical for transcription factor Krüpple like factor-2 (KLF-2) induction [57]. In addition, another study indicated that grape pomace extract (GPE) exerted antioxidant activity in endothelium (EA. hy926) through the increase of glutathione (GSH) levels due to increased gamma-glutamylcysteine synthetase (γ-GCS) levels and glutathione S-transferase (GST) activity [58]. Moreover, it was found that a low dose (1 μg/mL) of grape seed extract (GSE) potentiated the inhibitory action of HUVECs on platelet reactivity by about 10%, which accounted, at least partially, for the protective effects of grape products against CVDs. However, a high concentration (up to 10 μg gallic acid equivalent/mL) of GSE impaired endothelial cell proliferation in vitro [59].

#### 3.1.2. Decreasing Blood Lipids

Hyperlipidemia can lead to lipoprotein deposition inside the vessel wall, and induce oxidative stress and the formation of oxidized low-density lipoprotein (Ox-LDL), which plays a key role in the pathogenesis of atherosclerosis. The GSE possesses potent lipid-lowering and antioxidant properties, which are beneficial to the prevention of atherosclerosis [60]. A study showed that plasma triglycerides (TG) were attenuated by red grape consumption [61]. The hypolipidemic effect of grape seed procyanidin extract at low doses was studied in hamsters, and results suggested that 25 mg/kg of the extract decreased body weight, protected against fat accumulation, lowered plasma free fatty acid (FFA), and reduced lipid and TG accumulation in the mesenteric white adipose tissue (MWAT). In addition, the extract exerted these effects in part through the activation of both β-oxidation and the glycerolipid (GL)/FFA cycle, mainly in the retroperitoneal white adipose tissue (RWAT) [62]. High-density lipoproteins (HDL) are responsible for transporting 20–30% of the total plasma cholesterol from tissues to the liver, as vehicles for reversing cholesterol transport, which help prevent or even regress atherosclerosis [63]. A study indicated that grape polyphenols modulated the activity of plasma HDL enzymes in old and obese rats. The result showed that grape polyphenols increased HDL paraoxonase (PON) and lecithin-cholesterol acyltransferase (LCAT) activity, reduced cholesteryl ester transfer protein (CETP) activity, and restored the function of HDL [64].

#### 3.1.3. Decreasing Blood Pressure

The hypotensive effect of grape polyphenols has been detected in several studies [52,61]. Administration of GSPE markedly alleviated hypertension-induced arterial remodeling [51]. SHR were used to assess the anti-hypertensive effect of grape seed procyanidin extract. The results showed that the extract significantly decreased systolic and diastolic BP of SHR in a dose-dependent manner, and at the dose of 375 mg/kg, the decrease of both BP reached the maximum value. Moreover, the anti-hypertensive effect of the extract (375 mg/kg) in SHR was quite similar to that of Captopril (50 mg/kg), which has been considered as a very effective anti-hypertensive drug in clinical practice [9]. Another study suggested chronic administration of GSPE significantly blocked the BP increase in ouabain induced hypertensive rats model, and the improvement of the aortic NO production impaired by ouabain was the possible mechanisms involved [57]. Furthermore, a study investigated the anti-hypertensive effect and mechanism of red grape berry powder on rats with metabolic syndrome (MS). The study indicated that grape berry powder lowered BP via its ability of inhibiting ET-1 secretion and increasing eNOS levels of endothelium in a concentration-dependent manner [61].

#### 3.1.4. Suppressing Platelets Function

Platelets play a pivotal role in physiological hemostasis. However, enhanced platelets activation, adhesion, and aggregation aggravate the formation of arteriosclerotic plaques. A study in vitro revealed the potential protective effects of GSE on hemostasis under the condition of hyperhomocysteinemia by reducing the toxicity action of homocysteine (Hcy) and its most reactive form homocysteine thiolactone (HTL) in blood. In human platelets incubated with Hcy (100 µM) or HTL (1 µM), GSE decreased platelet adhesion to collagen and fibrinogen, the platelet aggregation, and O_2_^−^ production in platelets [65]. Additionally, a study in vitro indicated that 1 µg/mL GSE reduced platelet reactivity by about 10% due to the direct effect of its polyphenol contents on HUVECs [59].

#### 3.1.5. Alleviating Ischemia/Reperfusion Injury

A study investigated the cardio-protective effect of grape extracts rich in malvidin, an anthocyanin isolated from red grape skins, on isolated and Langendorff perfused rat heart. The result showed that malvidin elicited cardio-protective effect against ischemia/reperfusion (I/R) damages by activating the phosphatidylinositol 3-kinase (PI3K)/NO/cyclic guanosine monophosphate (cGMP)/protein kinase-G (PKG) pathway, increasing intracellular cGMP and the phosphorylation of eNOS, PI3K-AKT, extracellular regulated kinase1/2 (ERK1/2), and glycogen synthase kinase-3 β (GSK-3 β) [56]. In addition, grape extracts moderated cardiac and cerebral ischemia damages against I/R, which induced a drastic oxidative stress [56,66]. Moreover, a study investigated the relationship between grape seed and skin extract (GSSE) and ischemic stroke, and results showed that the extract not only reduced brain damage size and histology caused by I/R, but also inhibited oxidative stress, and improved transition metals associated enzyme activities [66]. Reperfusion arrhythmias (RA) are the most important causes of sudden death following reperfusion [67]. Another study analyzed the molecular mechanisms of protective effects of GSPE on RA. The study indicated that GSPE played an essential role in decreasing free radical generation for it increased the activity of Na^+^/K^+^-ATPase due to the upregulation of Na^+^/K^+^-ATPase α1 subunit [67].

#### 3.1.6. Inhibiting Thrombosis

The dysfunction of vessel endothelial cells and platelets are major risk factors in the formation of atherosclerotic plaque. For the antithrombotic effect of proanthocyanidins, a study revealed that GSPE decreased the length and weight of thrombus, protected the integrity of endothelium, reduced thrombogenesis-promoting factors P-selectin, von Willebrand factor (vWF), and cellular adhesion molecules (CAMs), increased thrombogenesis-demoting factors CD34, vascular endothelial growth factor receptor-2 (VEGFR-2), and ADAMTS13 (a disintegrin and metalloproteinase with a thrombospondin type one motif, member 13), and downregulated inflammatory cytokines interleukine (IL)-6, IL-8, and tumor necrosis factor-alpha (TNF-α). Thus, GSPE facilitated endothelial protection and inhibited platelet aggregation, inflammatory responses, and thrombus formation [68].

Collectively, the consumption of grapes or products derived from grapes might reduce the incidence of CVDs through correcting endothelial dysfunction, reducing blood lipids, anti-hypertension, inhibiting oxidative stress, improving platelet function, alleviating I/R damages, protecting myocardial function, anti-thrombosis, and resisting inflammation. These effects might be due to several phytochemicals, such as resveratrol, anthocyanin, and proanthocyanidin.

### 3.2. Blueberry

Blueberry is a flavonoid-containing fruit and exerts cardiovascular benefits. The cardioprotective effects of blueberry (*Vaccinium ashei* Reade) extract were investigated in hypercholesterolemic rats for 14 days. The result showed that blueberry extract decreased aortic lesions, reduced serum lipid profiles (total cholesterol (TC), low-density lipoprotein cholesterol (LDL-C), and TG), and increased activities of antioxidant enzymes (CAT, SOD, and glutathione peroxidase (GSH-Px)) [69]. The effects of supplementation with blueberry for 10 weeks on endothelial function and BP were studied in rats fed a high-fat diet. The study showed that blueberry supplementation lowered SBP by 14% and improved endothelial dysfunction and aorta relaxation in response to acetylcholine [70]. Furthermore, a study evaluated the potential protective effects of seven phenolic acids, identified as metabolites of blueberry, on murine macrophage cell line RAW 264.7. The result indicated that phenolic acids decreased foam cell formation induced by Ox-LDL, Ox-LDL binding to macrophages, lipopolysaccharide (LPS)-induced mRNA expression, and protein levels of TNF-α and IL-6 via inhibiting the phosphorylation of mitogen-activated protein kinase (MAPK), Jun N-terminal kinase (JNK), p38, and ERK1/2, downregulated the mRNA expression and protein levels of scavenger receptor CD36, and upregulated the mRNA expression and protein levels of ATP-binding cassette transporter A1 (ABCA1), which facilitated cholesterol efflux and inhibited cholesterol accumulation in macrophages [71].

In conclusion, blueberry possesses commendably cardioprotective ability including anti-atherogenic properties, anti-inflammation, lowering BP, improving oxidative parameters, and vascular reactivity.

### 3.3. Pomegranate

The peel, seed, and juice of pomegranate are rich in antioxidants and have potent atheroprotective effect and antihypertensive properties. The major bioactive constituent of pomegranate is punicalagin, which is known to have cardiovascular protective ability for its antioxidant role as a scavenger and ferrous chelator of hydrogen peroxide [72]. A study found that pomegranate extract (PE) reducing aortic sinus and coronary artery atherosclerosis was associated with the reduced oxidative stress and inflammation in the vessel wall of SR-BI/apoE double KO mice [73]. The high level of oxidative stress in the paraventricular nucleus of the hypothalamus is essential in the pathogenesis of hypertension. A study investigated the antihypertensive properties of PE in a SHR model. The findings demonstrated that PE alleviated hypertension by reducing oxidative stress, increasing the antioxidant defense system, decreasing inflammation, and improving mitochondrial function in the paraventricular nucleus, thereby activating AMPK-nuclear factor-erythroid 2 p45-related factor 2 (Nrf2) pathway [74]. Similarly, the activation of the AMPK pathway by PE was studied in the heart of a rodent obesity model. The result showed that PE activated AMPK by quickly decreasing the cellular ATP/ADP ratio specifically in cardiomyocytes, and the activation of the AMPK pathway accounted for the prevention of mitochondrial loss by enhancing mitochondrial biogenesis and amelioration of oxidative stress via increasing the activity of phase II enzymes in high-fat diet-induced cardiac metabolic disorders [72]. In addition, pomegranate seed extract improved motor and cognitive deficits due to permanent cerebral hypoperfusion ischemia (CHI), which was most likely related at least in some part to its antioxidant and free radical scavenging actions [75].

### 3.4. Apple

Apple is the second most consumed fruit in the world following banana. In recent years, epidemiological studies have shown that eating apples is associated with the reduction of the occurrence of CVDs [30,31]. Apple is a major source of fiber and contains antioxidants such as vitamin C and good dietary polyphenols. Particularly, the reduced incidence of CVDs is related to apple consumption, probably as a result of the cholesterol-lowering effect of polyphenols, the main bioactive compounds of apple, which are concentrated in the fruit peel. The cholesterol-lowering effect of apple was detected in male Wistar rats fed with a cholesterol-enriched diet (2%). The study showed that Bravo de Esmolfe apple was able to decrease serum levels of TG, TC, LDL-C, and Ox-LDL by 27.2%, 21.0%, 20.4%, and 20.0%, respectively. It also indicated that the cholesterol-lowering ability of apple was mainly due to phytocompounds, such as catechin, epicatechin, procyanidin B1, and β-carotene [76]. The development of CVDs is related with the previous existence of MS. Another study suggested that apple peel reduced the biochemical parameters (glycaemia, TC, high-density lipoprotein cholesterol (HDL-C), LDL-C, TG, ureic nitrogen, insulin, and asymmetric dimethylarginine (ADMA)) in CF-1 mice with MS, diminished the cholesterol accumulation area, and reverted the progression of the atherogenesis in apoE^−/−^ mice [77].

### 3.5. Hawthorn

Hawthorn (*Crataegus pinnatifida* Bge.) is a berry-like fruit from the species of *Crataegus*. It has been used as food or a traditional medicine to improve digestion for thousands of years. Moreover, during the last decades, hawthorn has received more attention because of its potential to treat CVDs, especially hyperlipidemia and atherosclerosis [78]. A study investigated the hypolipidemic effect of hawthorn fruit compounds (HFC, including hawthorn and kiwi fruit extract) in apoE^−/−^ atherosclerotic mice with high blood lipid levels. The study indicated that HFC reduced TG and LDL-C/TC ratio. Moreover, the reduction of LDL-C was more evident in HFC than in Simvastatin (6 mg/kg/day), indicating HFC could be considered for the treatment of hyperlipidemia and the prevention of atherosclerosis [10]. Similarly, hawthorn pectin pentaoligosaccharide (HPPS) suppressed weight gain, decreased serum TG levels, increased lipid excretion in feces, upregulated the gene and protein expressions of peroxisome proliferator-activated receptor α (PPAR-a), and enhanced the hepatic fatty acid oxidation-related enzyme activities of acyl-CoA oxidase, carnitine palmitoyltransferase I, 3-ketoacyl-CoA thiolase, and 2,4-dienoyl-CoA reductase by 53.8%, 74.2%, 47.1%, and 24.2%, respectively, in the liver of hyperlipidemic mice [79]. The anti-atherosclerosis effect of hawthorn and the potential mechanisms were investigated in apoE^−/−^ mice. The result showed that hawthorn decreased atherosclerotic lesions, serum TC and TG level, reduced the hepatic fatty acid synthase (FAS) and sterol regulatory element binding protein-1c (SREBP-1c) mRNA levels by 42% and 23%, and increased total antioxidant capacity (T-AOC), SOD and GSH-Px activities, and the mRNA expression levels of the antioxidant enzymes SOD1, SOD2, glutathione peroxidase-3 (Gpx3) in the livers of mice fed with hawthorn fruit diet [80]. Another study indicated that aqueous extract of hawthorn (*Crataegus pinnatifida* var. Major) inhibited atherosclerosis progression in high-fat-diet-fed rats by improving lipid metabolism, decreasing inflammatory cytokine responses, and protecting endothelium. The result showed that aqueous extract of hawthorn inhibited artery lesion, decreased IMT, reduced TC, TG, LDL-C, and the levels of CRP, IL-1β, IL-8, and IL-18, increased HDL-C, ET, 6-keto-prostaglandin F1α (6-keto-PGF1α), and thromboxane B2 (TXB2). It also revealed that chlorogenic acid, procyanidin B2, (−)-epicatechin, rutin, and isoquercitrin were the main components of the extract [81].

### 3.6. Avocado

Avocado is an essential tropical fruit containing lipophilic compounds, i.e., monounsaturated fatty acids (MUFAs), polyphenols, carotenoids, vitamin E, phytosterols, and squalene, which have been recognized for cholesterol-lowering ability [82]. However, the antioxidant capacities of these lipophilic compounds have attracted far less attention compared with hydrophilic compounds in the fruit. In fact, the lipophilic extract of the fruit had higher antioxidant capacity than its hydrophilic extract [83]. A study indicated that avocado pulp, containing acetogenin compounds, inhibited platelet aggregation with a potential preventive effect on thrombus formation [84]. Moreover, avocado pulp contains variable oil contents and is widely used in many fields such as the pharmaceutical industry [82]. Another study evaluated the effects of avocado oil administration on inflammatory and lipid parameters in rats with metabolic changes induced by sucrose ingestion. The study demonstrated that avocado oil reduced hs-CRP and TG, very low-density lipoprotein (VLDL), and LDL levels [85]. In addition, the protective effects of dietary consuming avocado oil on biochemical markers of liver function in rats fed with sucrose were quite similar to olive oil [86]. Furthermore, a study has shown that avocado seeds improved hypercholesterolemia, and facilitated the prevention and treatment of hypertension, inflammatory conditions, and diabetes [87].

### 3.7. Other Fruits

Mango is rich in several bioactive components with antioxidant and anti-inflammatory properties, such as carotenoids, vitamin C, and phenolic compounds. A study demonstrated that two doses (1% and 10%) of freeze-dried mango pulp were effective in improving glucose tolerance and lipid profiles and reducing adiposity in mice fed with a high-fat diet. Additionally, the study also reported that the lower dose (1%) was more effective in modulating glucose than the higher dose (10%), and was more powerful in lowering blood glucose concentration than the hypoglycemic drug, rosiglitazone (50 mg/kg diet), in mice fed with a high-fat diet [11]. Moreover, the anti-hypertensive effects of the standardized methanolic extract of papaya (*Carica papaya*) were evaluated in SHR. The result showed that the angiotensin converting enzyme inhibitory effects of papaya (100 mg/kg) were similar to those of enalapril (10 mg/kg). The flavonoids, especially quercetin, rutin, nicotiflorin, clitorin, and manghaslin, were identified as bioactive components of the extract, which could be applied to the treatment of hypertension [12]. In addition, several studies revealed that cherry, Guangzao (*Choerospondias axillaris*), and acai (*Euterpe oleracea* Mart.) have significant cardioprotective effects and have been shown to play a beneficial role in improving myocardial infarction induced by I/R via anti-oxidative and anti-apoptotic activities [88,89,90]. In addition, bilberry, black raspberry, and sea buckthorn berries improved serum lipid profiles and promoted a hypocholesterolemic effect, which protected against hypercholesterolemia and prevented atherosclerosis [91,92,93]. Additionally, jujube (*Zizyphus jujuba)* and blackberry (*Rubus allegheniensis* Port.) inhibited foam cell formation in human monocyte-derived macrophages induced by acetylated LDL, which therefore were useful for the prevention of atherosclerosis [94,95]. In addition, yellow passion fruit and boysenberry decreased BP in SHR [96,97]. However, data on these individual fruits is still limited. Furthermore, the underlying mechanisms of protecting cardiovascular system remain to be investigated.

**Figure 1:**
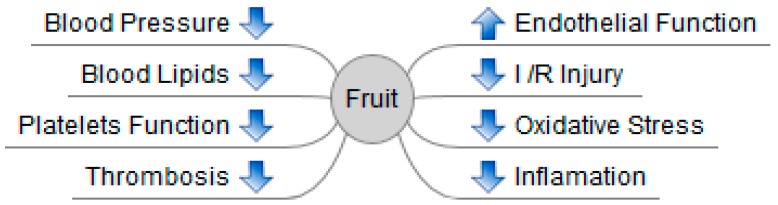
Effects and mechanisms of fruits on cardiovascular diseases (CVDs).

In conclusion, fruits such as grape, blueberry, pomegranate, apple, hawthorn, and avocado showed protective effects on cardiovascular function. Grape products markedly alleviated hypertension-induced cardiovascular remodeling and impaired endothelial function. Most fruits were effective in reducing oxidative stress, regulating lipids metabolism, and modulating BP. Additionally, some fruits attenuated platelet function, alleviated I/R injury, suppressed thrombosis, and inhibited inflammation (Figure 1).

## 4. Clinical Trials

The anti-hypertensive effect of grape polyphenols in several randomized controlled trials (RCTs) was evaluated by a meta-analysis, and results showed that daily grape polyphenols intake significantly reduced SBP by 1.48 mmHg when compared with control subjects (*p* = 0.03). Contrarily, DBP was not significantly decreased [108]. Grapes have potent hypolipidemic and anti-oxidative effects. Several studies showed that grape reduced TC, LDL-C, and Ox-LDL and increased HDL-C in subjects with various risk factors of CVDs [60,109,110]. Additionally, a study conducted on 60 healthy volunteers indicated that supplying them with 700 mg polyphenol-rich grape extracts for 56 days modulated the lipid profiles in terms of cardiovascular risk indicators, lowered TC and LDL-C, and increased antioxidant capacity and vitamin E [111]. Moreover, a meta-analysis of 9 RCTs explored the endothelium protective effect of grape polyphenols supplementation in adults. The study suggested that consuming grape polyphenols improved endothelial function in healthy subjects, and the effect was more obvious in subjects with high cardiovascular risk factors [112]. Besides grapes, other berries such as strawberry, acai (*Euterpe oleracea* Mart.), Caucasian whortleberry (*Vaccinium arctostaphylos* L.), sea buckthorn, and bilberry also have a potent lipid-lowering effect [113,114,115,116,117,118,119,120,121]. The benefits of berries on the serum lipid metabolism might contribute to anthocyanin. The effects of berry-derived anthocyanin supplements on the serum lipid profiles were studied in 120 dyslipidemic patients. The results suggested that anthocyanin intake increased HDL-C and cellular cholesterol efflux to serum, and decreased LDL-C, possibly due to the inhibition of CETP [122].

A clinical trial evaluated the cardiovascular protective effects of consumption of 75 g (about two medium-sized apples) of dried apple for 1 year in 146 postmenopausal women. The study showed that dried apple significantly lowered serum levels of TC and LDL-Cl by 9% and 16%, respectively, at 3 months and further decreased by 13% and 24%, respectively, at 6 months, but stayed constant thereafter. Furthermore, consumption of dried apple also reduced lipid hydroperoxide and CRP [123]. In addition, a study compared the cholesterol-lowering effect of 5 different apple species, Red Delicious, Granny Smith, Fuji, Golden Delicious and Annurca apple, in mildly hypercholesterolaemic healthy subjects. The study detected that Annurca apples led to the most significant outcome, reduced TC and LDL-C levels by 8.3% and 14.5%, respectively, and an increased HDL-C level by 15.2% (all *p* < 0.001) [124]. Moreover, another study compared the effects of whole fresh apple and processed apple products (apple pomace, cloudy apple juice, or clear apple juice) on lipid profiles in healthy volunteers. The result showed that whole apple, pomace, and cloudy juice lowered serum TC and LDL-C; however, clear apple juice increased TC and LDL-C slightly, from which it could be concluded that the fiber component was necessary for the lipid-lowering effect of apple in healthy humans [125]. Additionally, the acute effects of apple on improving endothelial function were studied in some trials, showing that apple improved endothelial function by affecting NO metabolites [126,127].

Kiwifruit is a good source of antioxidants due to its wealth in vitamins C and E, folate, carotenoids, and phytochemicals and protects the body from endogenous oxidative damage [128]. A clinical trial conducted on 85 hypercholesterolemic men showed that consuming two green kiwifruits daily in conjunction with a healthy diet reduced inflammatory markers and lipid profiles in subjects with modestly elevated CRP [129], but there were no significant differences in BP [130]. In addition, a study of 43 subjects who had hyperlipidemia indicated that regular consumption of kiwifruit not only modulated lipids profiles but also exerted beneficial effects on the antioxidant status via decreasing LDL oxidation and oxidative stress [131]. Moreover, another study conducted on 118 subjects with moderately elevated BP or stage 1 hypertension (SBP: 130–159 mmHg, DBP: 85–99 mmHg) showed that mean 24 h ambulatory systolic/diastolic BP was lower in the group consuming three kiwifruits versus the group consuming one apple daily [132]. The hypotensive effect of kiwifruit, to some extent, was more notable in individuals with moderately elevated BP. Furthermore, the beneficial effects of consuming three kiwifruits per day on BP and platelet aggregation were studied in male smokers. The resulted showed that kiwifruits reduced the SBP and DBP by 10 mmHg (*p* = 0.019) and 9 mmHg (*p* = 0.016), respectively, decreased platelet aggregation by 15% (*p* = 0.009), and lowered ACE activity by 11% (*p* = 0.034) [133].

Avocados are a nutrient-dense source of MUFAs that can be used to replace saturated fatty acids (SFA) in a diet to lower LDL-C. A meta-analysis of 10 RCTs assessing the impacts of avocados on TC, LDL-C, HDL-C, and TG revealed that avocado decreased TC, LDL-C, and TG levels by 18.80 mg/dL, 16.50 mg/dL, and 27.20 mg/dL, respectively [134].

Finally, results from clinical trials are summarized in Table 3. Numerous clinical trials have demonstrated that grape, apple, kiwifruit, and avocado were potential candidates for cardiovascular protection due to their potent lipid-lowering efficiency. However, clinical studies on other fruits are relatively few, and more research is needed to investigate the potential in combating CVDs.

## 5. Conclusions

The CVDs are greatly related to unbalanced diets. Several fruits can modulate metabolic risk factors such as hypertension, dyslipidemia, diabetes, and overweight/obesity, and inhibit atherosclerosis, which is the key pathological process of CHD and stroke. Many epidemiological studies investigating the relationship between fruit consumption and CVD risks yielded similar results regarding the protective effects of fruits on CVDs. Moreover, the majority of experimental studies also supported cardiovascular protecting properties of several fruits, such as grape, blueberry, pomegranate, apple, hawthorn, and avocado. The mechanisms of action mainly included the modulation of molecular events and signaling pathways associated with correcting endothelial dysfunction, reducing disorders in lipids metabolism, anti-hypertension, suppressing platelets function, alleviating I/R injury, inhibiting thrombosis, reducing oxidative stress, and inhibiting inflammation responses. In the future, the protective effects of a greater number of fruits on CVDs should be evaluated, and the bioactive components should be isolated and identified. Furthermore, the mechanisms of action should be further studied.

## Figures and Tables

**Table 1:** Fruit intake and CVD risk.

Subject	Study Type	Dose	Disease	Risk Estimates (95%CI)	References
512,891 Chinese adults (age: 30–79 years)	cohort study	daily vs. never/rarely fresh fruits	cardiovascular death	0.60 (0.54–0.67)	[4]
			incident major coronary events	0.66 (0.58–0.75)	
			ischemic stroke	0.75 (0.72–0.79)	
			hemorrhagic stroke	0.64 (0.56–0.74)	
30,458 UK Women (age: 35–69 years)	cohort study	per 80 g/day total fruits	CVD	0.94 (0.89–1.00)	[5]
			CHD	0.93 (0.85–1.01)	
		per 80 g/day fresh fruits	CVD	0.92 (0.85–1.00)	
			CHD	0.89 (0.79–1.00)	
1456 women (age: >70 years)	cohort study	per 129 g/day total fruits	CVD	NA (*p* < 0.05)	[6]
10,623 Japanese (4147 men, 6476 women)	cohort study	near-daily vs. infrequent citrus fruits	CVD	Men: 0.57 (0.33–1.01) Women: 0.51 (0.29–0.88)	[7]
67,211 women in Shanghai, China (age: 40–70 years)	cohort study	449 vs. 83 g/day fruits	CHD	0.62 (0.37, 1.03)	[28]
23 cohort studies of 937,665 participants and 18,047 patients with CHD	meta-analysis	the highest vs. the lowest of total fruits	CHD	0.86 (0.82–0.91)	[29]
		per 300 g/day fruits		0.84 (0.75–0.93)	
25,065 men in Denmark (age: 50–64 years)	cohort study	per 25 g/day apples	ACS	0.97 (0.94, 0.99)	[30]
74,961 Swedish adults (34,670 women, 40,291 men; age: 45–83 years)	cohort study	3.1 vs. 0.4 servings/day total fruits	total stroke	0.87 (0.78–0.97)	[31]
		1.0 vs. 0.1 servings/day apples/pears		0.89 (0.80–0.98)	
20,069 adults in the Netherlands (age: 20–65 years)	cohort study	>120 vs. ≤120 g/day raw fruits	hemorrhagic stroke	0.53 (0.28–1.01)	[33]
		per 25 g/day white fruits (usual apples and pears)	stroke	0.91 (0.85–0.97)	[32]
69,622 women from the Nurses’ Health Study	cohort study	the fifth vs. the lowest quintile of citrus fruits/juices	ischemic stroke	0.90 (0.77–1.05)	[34]
		the fifth vs. the lowest quintile of flavanone		0.81 (0.66–0.99)	
20,024 participants without stroke history	cohort study	the highest vs. the lowest quintile of citrus fruits/juices	ischemic stroke	0.69 (0.53–0.91)	[35]
		the highest vs. the lowest quintile of flavonoid		0.72 (0.55–0.95)	
28,082 US women (age: ≥39 years)	cohort study	≥3 vs. <0.5 servings/day total fruits	hypertension	0.89 (0.81–0.96)	[37]
745 residents from Ohasama, Japan without hypertension at baseline (age: ≥35 years)	cohort study	the highest vs. the lowest quartile of fruits	hypertension	0.40 (0.21–0.74)	[38]
3 large longitudinal cohort studies of 187,453 subjects	cohort study	≥4 vs. ≤4 servings/week of total whole fruits	hypertension	0.92 (0.87–0.97)	[39]
9791 subjects in Korea (3819 men, 5972 women )	case-control study	the fifth vs. the lowest quintile of fruits	hypertension	0.73 (0.61–0.88)	[40]
255 Chinese patients with type 2 diabetes (137 men, 118 women)	cross-sectional study	92.6 ± 39.7 vs. 14.5 ± 8.6 g/day fruits	carotid IMT (0.97 ± 0.02 vs. 1.08 ± 0.03 mm) prevalence of carotid plaque (1.18 vs. 11.76%)	NA (*p* = 0.046) NA (*p* = 0.022)	[41]
407 patients with type 2 diabetes (172 men, 235 women)	cross-sectional study	101.3 ± 28.5 vs. 79.6 ± 24.2g/day fruits	carotid IMT	0.92 (0.67–0.95)	[42]
			hs-CRP	0.69 (0.53–0.89)	
43,880 healthy men who had no prior diagnosed CVDs or cancer	cohort study	higher anthocyanin intake	MI	0.87 (0.75–1.00)	[43]
		higher flavanone intake	ischemic stroke	0.78 (0.62–0.97)	
1393 Chinese adults	cross-sectional study	higher anthocyanins intake	HDL-C	NA (*p* = 0.001) (women)	[44]
		higher total flavonoid intake	TG	NA (*p* = 0.020) (women)	
			TG/HDL-C ratios	NA (*p* = 0.040) (women)	
		higher flavonol intake	TG	NA (*p* = 0.035) (women)	
			TG/HDL-C ratios	NA (*p* = 0.045) (women)	
29,689 Italian women	cohort study	the highest vs. the lowest quartile of fruits	CHD	no significant association	[45]
8060 men aged 50–59 years in France and Northern Ireland	cohort study	≥1.29 vs. ≤0.57 times/day fruits	ACS	no significant association	[46]
164,617 men and women from five ethnic groups	cohort study	>4.9 vs. <1.5 servings/day fruits	ischemic heart disease	no significant association	[47,48]
34,319 Swedish women aged 49–83 years	cohort study	≥2.6 vs. ≤0.8 servings/day total fruits	heart failure	no significant association	[49]

NA, stands for not available.

**Table 2:** The cardioprotective abilities of fruits.

Fruit	Subject	Study Type	Dose	Main Effects	References
**Grape**					
freeze-dried grape powder	SHR and Wistar-Kyoto (WKY) rats	in vivo	600 mg/day	BP↓, arterial relaxation↑, vascular compliance↑, cardiac hypertrophy↓	[51]
GSPE	SHR	in vivo	250 mg/kg/day	arterial remodeling↓, ET-1↓, NO↑, SOD↑, CAT↑, MDA↓	[52]
oligomeric grape seed proanthocyanidins (GSPs)	mice treated with DOCA-salt to induce cardiovascular remodeling	in vivo	NA	heart weight/body weight ratio↓, kidney weight/body weight atio↓, cross-sectional area of cardiomyocytes↓, collagen deposition in heart↓, histopathology injury↓, NO↑, SOD↑, MDA↓	[53]
	isolated thoracic aorta ring	in vitro		endothelial-dependent aorta ring relaxation↑	
GP-EE	rat aorta and small mesenteric artery (SMA) segments	in vitro	0.3 and 10 μM	endothelium- and NO-dependent vasodilatation↑, phenylephrine(Phe)-induced response in aortic rings↓, O_2_^−^↓, contraction elicited by ET-1↓	[54]
red grape skin and seeds polyphenols	human endothelial progenitor cells (EPC)	in vitro	5, 50 and 150 µg/mL	EPC viability and function↑, endothelial dysfunction↓, hyperglycemia effect↓, ROS production↓	[55]
GSPE	ouabain induced hypertensive rats model	in vivo	250 mg/kg/day	BP↓, aortic NO production↑	[57]
	HUVECs	in vitro	10 µg/mL	eNOS expression↑	
GPE	endothelial (EA. hy926) cells	in vitro	0.068 and 0.250 µg/mL	GCS levels↑, GST activity↑, antioxidant activity↑	[58]
GSE	HUVECs	in vitro	1 μg/mL	platelet reactivity↓	[59]
red grape berry powder	rats with metabolic syndrome	in vivo	200, 400 and 800 mg/kg/day	BP↓, plasma TG↓, insulin↓	[61]
	HUVECs	in vitro	20–1400 μg/mL	ET-1↓	
			0.011, 0.058, 0.29, 1.46 and 3.66 mg/mL	eNOS level↑	
grape seed procyanidin extract	hamster	in vivo	25 mg/kg/day	body weight gain↓, adiposity index↓, weight of white adipose tissue depots↓, plasma phospholipids↓, plasma FFA↓, mesenteric lipid and triglyceride accumulation↓	[62]
grape polyphenols from *Vitis vinifera* grapes	24-month-old obese rats	in vivo	90 mg/kg/day	plasma HDL PON activity↑, LCAT activity↑, CETP activity↓	[64]
grape seed procyanidin extract	SHR	in vivo	375 mg/kg	SBP↓, DBP↓, GSH activity↑	[9]
GSE or black chokeberry (*Aronia melanocarpa*) extract	human platelets incubated with Hcy (100 µM) or HTL (1 µM)	in vitro	2.5, 5, 10 µg/mL	platelet adhesion to collagen and fibrinogen↓, platelet aggregation↓, O_2_^•−^ production in platelet↓	[65]
malvidin-rich red grape skin extract	isolated and Langendorff perfused rat heart	in vitro	1–1000 ng/mL	I/R damages↓, coronary dilation↑, active PI3K/NO/cGMP/PKG pathway, intracellular cGMP↑, eNOS, PI3K-AKT, ERK1/2, and GSK-3 β phosphorylation↑	[56]
GSSE	a rat model of global ischemia	in vivo	2.5 g/kg	brain damage size and histology↓, oxidative stress↓, transition metals associated enzyme activities↑	[66]
GSPE	isolated rat hearts	in vitro	NA	RA↓, Na^+^/K^+^-ATPase activity↑, Na^+^/K^+^-ATPase α1 subunit↑, free radical↓	[67]
GSPE	a rat model of deep vein thrombosis (DVT)	in vivo	400 mg/kg/day	thrombus length and weight↓, protecte endothelium integrity, IL-6, IL-8 and TNF-α↓	[68]
**Blueberry**					
blueberry extract (*Vaccinium ashei* Reade)	hypercholesterolemic rat	in vivo	25, 50 mg/kg	aortic lesions↓, oxidative damage to lipids and proteins↓, TC↓, LDL-C↓, TG↓, activity of CAT, SOD and GSH-Px↑	[69]
freeze-dried blueberry powder	rats fed a high-fat/cholesterol diet	in vivo	2% (*w*/*w*)	SBP↓, aorta relaxation↑, endothelial dysfunction↓	[70]
7 phenolic acids of freeze-dried blueberry	murine macrophage cell line RAW 264.7	in vitro	NA	TNF-α and IL-6 mRNA expression and protein levels↓, MAPK, JNK, p38, and Erk1/2 phosphorylation↓, mRNA expression and protein levels of scavenger receptor CD36↓, foam cell formation↓, expression and protein levels of ABCA1↑	[71]
**Pomegranate**					
PE	SR-BI/apoE double KO mice	in vivo	307.5 µL/L in water	aortic sinus and coronary artery atherosclerosis↓, oxidative stress and inflammation in the vessel wall↓	[73]
PE containing 40% punicalagin	SHR	in vivo	150 mg/kg/day	BP↓, cardiac hypertrophy↓, oxidative stress↓, antioxidant defense system↑, paraventricular nucleus inflammation↓, mitochondrial superoxide anion levels↓, mitochondrial function↑	[74]
PE containing 40% punicalagin	heart of a high-fat diet-induced obesity rat model	in vivo	150 mg/kg/day	mitochondrial biogenesis↑, oxidative stress↓, phase II enzymes↑, cardiac metabolic disorders↓	[72]
pomegranate seed extract	CHI rat model	in vivo	100, 200, 400, 800 mg/kg/day	motor and cognitive coordination↑	[75]
**Apple**					
Bravo de Esmolfe apple	male Wistar rats fed a cholesterol-enriched diet (+2% cholesterol)	in vivo	20% (*w*/*w*) = 5g/rat/day (~2–3 apples/person/day) for 30 days	serum TG↓, TC↓, LDL-C↓, oxLDL↓	[76]
Fuji apple peel Granny Smith apple peel	CF-1 mice with MS apoE^−/−^ mice	in vivo	20% (*w*/*w*) for 43 days 20% (*w*/*w*) for 10 weeks	glycaemia↓, TC↓, HDL-C↓, LDL-C↓, ureic nitrogen↓, TG↓, insulin↓, ADMA↓ atherogenic progression↓, cholesterol accumulation area↓	[77]
**Hawthorn**					
HFC	apoE^−/−^ atherosclerotic mice with high blood lipid levels fed with a high cholesterol diet	in vivo	0.5 mL/day	TG↓, LDL-C/TC ratio↓	[10]
HPPS	the liver of high fat diet induced hyperlipidemic mice	in vivo	150 mg/kg	weight gain↓, TG↓, lipid excretion in feces↑, mRNAs and activities of acyl-CoA oxidase, carnitine palmitoyltransferase I, 3-ketoacyl-CoA thiolase, and 2,4-dienoyl-CoA reductase↑, gene and protein expressions of PPAR-α↑	[79]
freeze dried hawthorn fruit (*Crataegus pinnatifida)*	apoE^−/−^ mice	in vivo	1% (*w*/*w*)	atherosclerotic lesions↓, TC↓, TG↓, T-AOC values↑, SOD and GSH-Px activities↑, hepatic FAS and SREBP-1c mRNA levels↓, hepatic SOD1, SOD2, Gpx3 mRNA levels↑	[80]
sugar-free aqueous extract of hawthorn fruit (*Crataegus pinnatifida* var. Major)	high fat diet fed rats	in vivo	72 and 288 mg/kg/day	TC, TG and LDL-C↓, HDL-C↑, CRP, IL-1β, IL-8 and IL-18↓, ET, 6-keto-PGF1α and TXB2↑, pathological changes in the arteries↓, IMT↓	[81]
**Avocado**					
avocado pulp (*Persea americana*) extract	male adult CD 1 mice	in vivo	25 mg/kg	thrombus formation↓	[84]
	platelet	in vitro	10 µL	platelet aggregation↓	
avocado oil	rats ingested with sucrose	in vivo	7.5% (*w*/*w*)	TG↓, VLDL↓, LDL↓, hs-CRP↓	[85]
**Others**					
freeze-dried mango pulp	male C57BL/6J mice fed a high-fat diet	in vivo	1% or 10% (*w*/*w*)	epididymal fat mass↓, percentage of body fat↓, improve glucose tolerance, insulin resistance↓	[11]
methanolic extract of papaya (*Carica papaya*)	SHR	in vivo	100 mg/kg (twice a day)	BP↓, angiotensin converting enzyme(ACE) activity↓, cardiac hypertrophy↓, improve baroreflex sensitivity	[12]
sour cherry seed kernel extract	hearts from Sprague-Dawley rats	in vitro	30 mg/kg/day	post ischemic cardiac functions↑, infarct size↓, heme oxygenase-1 (HO-1)↑, Bcl-2↑	[88]
total flavonoids of Guangzao (*Choerospondias axillaris*)	I/R male Sprague-Dawley rats	in vivo	75, 150 and 300 mg/kg/day	cardiac function↑, heart pathologic lesion↓, CAT↑, GSH-Px↑, SOD↑, MDA↓, TUNEL-positive nuclear staining↓, Bcl-2-associated X protein (Bax)↓, caspase-3↓, Bcl-2↑, p38 MAPK activity↓, JNK activity↓	[89]
hydroalcoholic extract of acai (*Euterpe oleracea* Mart.) seeds	male Wistar rats subjected to myocardial infarction	in vivo	100 mg/kg/day	prevent the development of exercise intolerance, cardiac hypertrophy, fibrosis, and dysfunction	[90]
acai pulp	female Fischer rat of dietary-induced hypercholesterolemia	in vivo	2% (*w*/*w*)	TC↓, LDL-C↓, atherogenic index↓, HDL-C↑, cholesterol excretion in feces↑, expression of the LDL-R, ABCG5, and ABCG8 genes↑	[98]
bilberry (*Vaccinium myrtillus* L.) anthocyanin-rich extract	apoE^−/−^ mice	in vivo	0.02% (*w*/*w*)	improve hypercholesterolemia against atherosclerosis	[91]
unrefined black raspberry seed oils	male Syrian hamsters fed high-cholesterol (0.12%), high-fat (9%) diets	in vivo	NA	plasma and liver TG↓, hypertriglyceridemia↓	[92]
polyphenols from sea buckthorn berry	rats with hyperlipidemia	in vivo	7–28 mg/kg	serum lipids↓, TNF-α↓, IL-6↓, antioxidant enzymes activity↑, eNOS, ICAM-1, and LOX-1 mRNA expression and proteins in aortas↓	[93]
Jujube *(Zizyphus jujuba)* fructus and semen extract	human macrophages	in vitro	NA	the foam cell formation induced by acetylated LDL↓, prevent atherosclerosis	[94]
methanol extract of blackberry (*Rubus allegheniensis* Port.)	human monocyte-derived macrophages induced by acetylated LDL	in vitro	50 μM	foam cell formation↓	[95]
yellow passion fruit pulp	SHR	in vivo	5, 6 or 8 g/kg/day	SBP↓, GSH↑, thiobarbituric acid-reactive substances (TBARS)↓	[96]
proanthocyanidins in boysenberry seed extract	SHR	in vivo	100 and 200 mg/kg	SBP↓	[97]
	rat aorta rings	in vitro		vasorelaxant activity↑	
methanolic extract of date palm (*Phoenix dactylifera* L.)	cerebral ischemia rats	in vivo	100, 300 mg/kg	SOD↑, CAT↑, GSH↑, glutathione reductase↑, lipid peroxidation↓, oxidative stress↓, neuronal damage↓	[99]
black chokeberry (*Aronia melanocarpa*) extract	bovine coronary artery endothelial cells	in vitro	0.1 g/mL	NO↑, eNOS phosphorylation↑	[100]
saskatoon berry powder	leptin receptor-deficient diabetic mice	in vivo	5% (*w*/*w*)	monocyte adhesion to aorta↓, inflammatory, fibrinolytic or stress regulators in aorta or heart apex↓	[101]
saskatoon berry powder	leptin receptor-deficient diabetic mice	in vivo	5% (*w*/*w*)	endoplasmic reticulum stress (ERS)↓, unfolded protein response (UPR)↓	[102]
	glycated LDL-treated HUVECs	in vitro			
19 fruits widely consumed in central Chile	NA	in vitro	1 mg/mL	anticoagulant activities: grape, raspberry fibrinolytic activity: raspberry	[103]
peach (*Prunus persica*) pulp ethylacetate extract	cultured vascular smooth muscle cells (VSMCs)	in vitro	50, 100, or 200 µg/mL	Angiotensin II (Ang II) induced intracellular Ca^2+^ elevation↓, generation of ROS↓	[104]
methanolic extract of Lingonberry (*Vaccinium vitis-idaea* L.)	H9c2 rat myoblasts simulated IR	in vitro	5 and 10 µM	apoptosis↓, markers of nuclei condensation , caspase-3 activation, and MAPK signaling↓	[105]
blueberry anthocyanin fraction (BBA), blackberry anthocyanin fraction (BKA), and blackcurrant anthocyanin fraction (BCA)	RAW 264.7 macrophages treated by LPS bone marrow-derived macrophages from Nrf2^+/+^ mice treated by LPS	in vitro	0–20 μg/mL	IL-1 β mRNA levels↓, NF-κB p65 translocation to the nucleus↓ cellular ROS levels↓, IL-1β mRNA levels↓	[106]
pomegranate juice, together with date fruit and date seeds extract	apoE^−/−^ mice	in vivo	0.5 µM gallic acid equivalents (GAE)/day	TC↓, TG↓, PON1 activity↑, mouse peritoneal macrophage (MPM) oxidative stress↓, MPM cholesterol content↓, and MPM LDL uptake↓, aortas lipid peroxide content↓, aortas PON lactonase activity↑	[107]

NA, stands for not available.

**Table 3:** Clinical trials of fruits against CVDs.

Subject	Component	Treatment	Duration	Outcome	References
152 patients with type 2 diabetes	low glycaemic index fruit	−3.1 to 2.7 servings/day	6 months	HbA1c1↓, SBP↓, CHD risk↓	[135]
52 patients with mild hyperlipidemia	red grape seed extract (RGSE)	200 mg/day	8 weeks	TC↓, LDL-C↓, Ox-LDL↓	[60]
24 pre-hypertensive, overweight, and/or pre-diabetic subjects	whole grape extract (WGE)	350 mg/day	6 weeks	SOD↓, 8-isoprostane↓, Ox-LDL↓, TC/HDL-C ratios↓, HDL-C ↑	[109]
69 patients with hyperlipidemia	Condori red grapes or Shahroodi white grapes	500 g/day	8 weeks	thiobarbituric acid reactive substances (TBARS)↓, total antioxidant capacity (TAC)↑, TC↓, LDL-C↓	[110]
60 healthy volunteers	polyphenol-rich grape extract supplementation	700 mg/day	56 days	TC↓, LDL-C↑, TAC↑, vitamin E↑	[111]
96 women aged 40–60 years who had at least one menopausal symptom	grape seed extract tablets	=100 or 200 mg proanthocyanidin/day	4 weeks	SBP↓, DBP↓	[136]
70 untreated subjects with pre- and stage I hypertension (SBP: 120–159 mmHg)	grape seed extract (GSE) rich in low-molecular-weight polyphenolic compounds	300 mg/day	8 weeks	BP values were modestly, but not significantly, affected	[137]
75 patients at high risk of CVD (with diabetes or hypercholesterolemia plus ≥1 other CV risk factor) and undergoing primary prevention of CVDs	resveratrol-rich grape supplementation	350 mg/day = 8 mg resveratrol for the first 6 months and a double dose for the next 6 months	12 months	hs-CRP↓, TNF-á↓, plasminogen activator inhibitor type 1 (PAI-1)↓, IL-6/IL-10 ratio↓, IL-10↑	[138]
75 stable patients with CHD treated according to currently accepted guidelines for secondary prevention of CVDs	resveratrol-rich grape supplementation	350 mg/day = 8 mg resveratrol for the first 6 months and a double dose for the next 6 months	12 months	serum adiponectin↑, PAI-1↓, inflammatory genes in peripheral blood mononuclear cells (PBMCs)	[139]
48 participants with MS (4 men, 44 women; BMI: 37.8 ± 2.3 kg/m^2^; age: 50.0 ± 3.0 years)	freeze-dried blueberry	50 g(~350 g fresh)/day	8 weeks	SBP↓, DBP↓, Ox-LDL↓, MDA↓, serum hydroxynonenal↓	[140]
58 postmenopausal women with pre-andstage 1-hypertension	freeze-dried blueberry powder	22 g/day	8 weeks	SBP↓, DBP↓, brachial-ankle pulse wave velocity↓, NO↑	[141]
25 sedentary men and postmenopausal women (age: 18–50 years)	whole blueberry powder	~250 g berries/day	6 weeks	natural killer(NK) cells↑, augmentation index (AIx)↓, aortic systolic pressures (ASPs)↓, diastolic pressures↓	[142]
18 male volunteers (age: 47.8 ± 9.7 years; BMI: 24.8 ± 2.6 kg/m^2^)	freeze-dried wild blueberries (*Vaccinium angustifolium*) powder	25 g = 375 mg anthocyanins	6 weeks	endogenously oxidized DNA bases↓, H_2_O_2_-induced DNA damage↓	[143]
23 healthy subjects (11 men, 12 women; age: 27 ± 3.2 years; weight: 63.5 ± 12.7 kg; BMI: 21.74 ± 2.5 kg/m^2^)	strawberries	500 g/day	1 month	TC↓, LDL-C↓, TG↓, MDA↓, urinary 8-OHdG↓, isoprostanes↓, TAC↑, spontaneous and oxidative hemolysis↓, activated platelets↓	[113]
60 volunteers (5 men, 55 women; age: 49 ± 10 years; BMI: 36 ± 5 kg/m^2^)	freeze-dried strawberries (FDS)	25 or 50 g/day	12 weeks	TC↓, LDL-C↓, MDA↓	[114]
27 subjects with MS (2 men, 25 women; age: 47.0 ± 3.0 years; BMI: 37.5 ± 2.15 kg/m^2^)	FDS	50g (~3 cups fresh)/day	8 weeks	TC↓, LDL-C↓, small LDL particles↓, vascular cell adhesion molecule-1(VCAM-1)↓	[115]
36 subjects with type 2 diabetes (13 men, 23 women; age: 51.57 ± 10 years; BMI: 27.90 ± 3.7 kg/m^2^ )	FDS	50 g (~500 g fresh)/day	6 weeks	CRP↓, MDA↓, HbA1c↓, TAC↑	[144]
24 overweight and obese subjects (10 men, 14 women; age: 50.9 ± 15 years; ,BMI: 29.2 ± 2.3 kg/m^2^) consumed high carbohydrate/fat meal	strawberry (Fragaria) beverage	=10 g FDS (~100 g fresh)/day	6 weeks	TG↓, Ox-LDL↓, PAI-1↓, IL-1 β↓	[116,117]
10 overweight adults (BMI: 25–30 kg/m^2^)	acai pulp (*Euterpe oleracea* Mart.)	100 g twice/day	1 month	fasting glucose↓, postprandial plasma glucose↓, insulin↓, TC↓, LDL-C↓, TC/HDL-C ratio↓	[118]
23 healthy male volunteers (age: 30–65 years; BMI: 25–30 kg/m^2^)	acai-based smoothie	=694 mg total phenolics	1 d	flow-mediated dilatation (FMD)↑	[145]
72 dyslipidemic patients	blackberry (*Morus nigra* L.) juice with pulp	300 mL/day	8 weeks	apo A-I↑, HDL↑, apo B↓, hs-CRP↓, SBP↓	[146]
40 hyperlipidemic patients (age: 20–60 years)	Caucasian whortleberry (*Vaccinium arctostaphylos* L.) fruit hydroalcoholic extract	350 mg/8 h	2 months	TC↓, TG↓, LDL-C↓, HDL-C↑	[119]
80 overweight and obese female volunteers (BMI: 29.6 ± 2.1 kg/m^2^)	sea buckthorn berries (SB) sea buckthorn oil (SBo) SB phenolic extract (SBe) bilberries (BB)	~100 g/day fresh berries	33–35 days	SB: TG and VLDL↓, waist circumference↓; SBo: total lipoprotein, intermediate-density lipoprotein (IDL), LDL and LDL-C↓, vascular cell adhesion molecule (VCAM)↓; SBe: VLDL fractions and serum TG↑, intercellular adhesion molecule (ICAM)↓; BB: improve serum lipids and lipoproteins, waist circumference↓,body weight↓, VCAM↓	[120,121]
120 dyslipidemic subjects (age: 40–65 years)	berry-derived anthocyanin	160 mg twice/day	12 weeks	LDL-C↓, HDL-C↑, cellular cholesterol efflux to serum↑, mass and activity of plasma CETP ↓	[122]
160 postmenopausal women	dried apple	75 g/day	1 year	TC↓, LDL-C↓, lipid hydroperoxide↓, CRP↓	[123]
50 mildly hypercholesterolaemic healthy subjects (28 men, 22 women)	Annurca apple (*Malus pumila* Miller cv. Annurca)	2/day	4 months	TC↓, LDL-C↓, HDL-C↑	[124]
23 healthy volunteers	whole apples	550 g/day	4 weeks	whole apple, pomace and cloudy juice lowered serum TC and LDL-C	[125]
	apple pomace	22 g/day			
	clear apple juices	500 mL/day			
	cloudy apple juices	500 mL/day			
51 healthy adults (age: 40–60 years)	apple	1/day	4 weeks	Ox-LDL/β_2_-glycoprotein I complex (Ox-LDL-β_2_ GPI)	[147]
20 subjects (age: 21–29 years)	apple juice	two glasses (2 × 250 mL/day)	4 weeks	plasma antioxidant activity (FRAP)↑, insulin↑, HOMA↑, total GSH↓	[148]
30 healthy subjects (6 men, 24 women; age: 47.3 ± 13.6 years)	flavonoid-rich apple	120 g flesh + 80 g skin twice/day	1 d	NO status↑, endothelial function↑, FMD↑, pulse pressure↓, SBP↓	[126]
14 subjects (age: 45–70 years)	drink containing epicatechin from an apple extract	=140 mg epicatechin/day	1 d	NO metabolites	[127]
30 hypercholesterolemic volunteers	polyphenol-rich apple	40 g = 1.43 polyphenols/day	4 weeks	did not improve vascular function	[149]
85 hypercholesterolemic men consumed a healthy diet	green kiwifruit	2/day	8 weeks	plasma HDL-C↑, TC/HDL-C ratio↓, hs-CRP↓, IL-6↓	[129]
				did not improve BP and markers of cardiovascular function	[130]
43 subjects who had hyperlipidemia in Taiwan (13 men, 30 women)	kiwifruit	2/day	8 weeks	HDL-C↑, LDL-C/HDL-C ratio↓, TC/HDL-C ratio↓, vitamin C↑, vitamin E↑, LDL oxidation↓, MDA↓, 4-hydroxy-2-nonenal↓	[131]
118 subjects with moderately elevated BP or stage 1 hypertension (SBP: 130–159 mmHg, DBP: 85–99 mmHg)	kiwifruit	3/day	8 weeks	24-h ambulatory BP↓	[132]
102 male smokers (age: 44–74 years)	kiwifruit	3/day	8 weeks	SBP↓, DBP↓, platelet aggregation↓, ACE activity↓	[133]
45 overweight or obese participants with baseline LDL-C in the 25–90%	fresh Hass avocado	1(~36 g)/day	5 weeks	LDL-C↓, LDL-particle number↓, small dense LDL-C↓, LDL-C/HDL-C ratio↓	[150]
74 overweight adults	fresh Rio-Red grapefruit	0.5 with each meal (3x)/day	6 weeks	waist circumference↓, SBP↓, TC↓, LDL↓	[151]
12 obese postmenopausal women (age: 57 ± 1 years; BMI: 38.1 ± 2.1 kg/m^2^; SBP: 153 ± 4 mmHg)	L-citrulline-rich watermelon supplementation	=6 g L-citrulline/day	6 weeks	arterial stiffness↓, aortic SBP↓, pressure wave reflection amplitude↓	[152]

## References

[1] WHO Cardiovascular Diseases (CVDs). http://www.who.int/cardiovascular_diseases/en/.

[2] Rodríguez-Monforte M., Flores-Mateo G., Sánchez E. (2015). Dietary patterns and CVD: A systematic review and meta-analysis of observational studies. Br. J. Nutr..

[3] Grosso G., Marventano S., Yang J., Micek A., Pajak A., Scalfi L., Galvano F., Kales S.N. (2017). A comprehensive meta-analysis on evidence of Mediterranean diet and cardiovascular disease: Are individual components equal?. Crit. Rev. Food Sci. Nutr..

[4] Du H.D., Li L.M., Bennett D., Guo Y., Key T.J., Bian Z., Sherliker P., Gao H.Y., Chen Y.P., Yang L. (2016). Fresh fruit consumption and major cardiovascular disease in China. N. Engl. J. Med..

[5] Lai H.T.M., Threapleton D.E., Day A.J., Williamson G., Cade J.E., Burley V.J. (2015). Fruit intake and cardiovascular disease mortality in the UK Women’s Cohort Study. Eur. J. Epidemiol..

[6] Hodgson J.M., Prince R.L., Woodman R.J., Bondonno C.P., Ivey K.L., Bondonno N., Rimm E.B., Ward N.C., Croft K.D., Lewis J.R. (2016). Apple intake is inversely associated with all-cause and disease-specific mortality in elderly women. Br. J. Nutr..

[7] Yamada T., Hayasaka S., Shibata Y., Ojima T., Saegusa T., Gotoh T., Ishikawa S., Nakamura Y., Kayaba K. (2011). Frequency of citrus fruit intake is associated with the incidence of cardiovascular disease: The jichi medical school cohort study. J. Epidemiol..

[8] Ezzati M., Riboli E. (2013). Behavioral and dietary risk factors for noncommunicable diseases. N. Engl. J. Med..

[9] Quinones M., Guerrero L., Suarez M., Pons Z., Aleixandre A., Arola L., Muguerza B. (2013). Low-molecular procyanidin rich grape seed extract exerts antihypertensive effect in males spontaneously hypertensive rats. Food Res. Int..

[10] Xu H., Xu H.E., Ryan D. (2009). A study of the comparative effects of hawthorn fruit compound and simvastatin on lowering blood lipid levels. Am. J. Chin. Med..

[11] Lucas E.A., Li W.J., Peterson S.K., Brown A., Kuvibidila S., Perkins-Veazie P., Clarke S.L., Smith B.J. (2011). Mango modulates body fat and plasma glucose and lipids in mice fed a high-fat diet. Br. J. Nutr..

[12] Brasil G.A., Ronchi S.N., Do Nascimento A.M., de Lima E.M., Romao W., Da Costa H.B., Scherer R., Ventura J.A., Lenz D., Bissoli N.S. (2014). Antihypertensive effect of carica papaya via a reduction in ACE activity and improved baroreflex. Planta Med..

[13] Fu L., Xu B.T., Xu X.R., Gan R.Y., Zhang Y., Xia E.Q., Li H.B. (2011). Antioxidant capacities and total phenolic contents of 62 fruits. Food Chem..

[14] Zhang Y.J., Gan R.Y., Li S., Zhou Y., Li A.N., Xu D.P., Li H.B. (2015). Antioxidant phytochemicals for the prevention and treatment of chronic diseases. Molecules.

[15] Song F.L., Gan R.Y., Zhang Y., Xiao Q., Kuang L., Li H.B. (2010). Total phenolic contents and antioxidant capacities of selected chinese medicinal plants. Int. J. Mol. Sci..

[16] Gan R.Y., Xu X.R., Song F.L., Kuang L., Li H.B. (2010). Antioxidant activity and total phenolic content of medicinal plants associated with prevention and treatment of cardiovascular and cerebrovascular diseases. J. Med. Plants Res..

[17] Fu L., Xu B.T., Gan R.Y., Zhang Y., Xu X.R., Xia E.Q., Li H.B. (2011). Total phenolic contents and antioxidant capacities of herbal and tea infusions. Int. J. Mol. Sci..

[18] Guo Y.J., Deng G.F., Xu X.R., Wu S., Li S., Xia E.Q., Li F., Chen F., Ling W.H., Li H.B. (2012). Antioxidant capacities, phenolic compounds and polysaccharide contents of 49 edible macro-fungi. Food Funct..

[19] Deng G.F., Xu X.R., Zhang Y., Li D., Gan R.Y., Li H.B. (2013). Phenolic compounds and bioactivities of pigmented rice. Crit. Rev. Food Sci..

[20] Deng G.F., Lin X., Xu X.R., Gao L.L., Xie J.F., Li H.B. (2013). Antioxidant capacities and total phenolic contents of 56 vegetables. J. Funct. Foods.

[21] Li S., Li S.K., Gan R.Y., Song F.L., Kuang L., Li H.B. (2013). Antioxidant capacities and total phenolic contents of infusions from 223 medicinal plants. Ind. Crops Prod..

[22] Li A.N., Li S., Li H.B., Xu D.P., Xu X.R., Chen F. (2014). Total phenolic contents and antioxidant capacities of 51 edible and wild flowers. J. Funct. Foods.

[23] Xu D.P., Li Y., Meng X., Zhou T., Zhou Y., Zheng J., Zhang J.J., Li H.B. (2017). Natural antioxidants in foods and medicinal plants: Extraction, assessment and resources. Int. J. Mol. Sci..

[24] Fu L., Xu B.T., Xu X.R., Qin X.S., Gan R.Y., Li H.B. (2010). Antioxidant capacities and total phenolic contents of 56 wild fruits from South China. Molecules.

[25] Manach C., Scalbert A., Morand C., Remesy C., Jimenez L. (2004). Polyphenols: Food sources and bioavailability. Am. J. Clin. Nutr..

[26] Ginter E., Simko V. (2012). Plant polyphenols in prevention of heart disease. Bratisl. Med. J..

[27] Li A.N., Li S., Zhang Y.J., Xu X.R., Chen Y.M., Li H.B. (2014). Resources and biological activities of natural polyphenols. Nutrients.

[28] Yu D., Zhang X., Gao Y.T., Li H., Yang G., Huang J., Zheng W., Xiang Y.B., Shu X.O. (2014). Fruit and vegetable intake and risk of CHD: Results from prospective cohort studies of Chinese adults in Shanghai. Br. J. Nutr..

[29] Gan Y., Tong X., Li L., Cao S., Yin X., Gao C., Herath C., Li W., Jin Z., Chen Y. (2015). Consumption of fruit and vegetable and risk of coronary heart disease: A meta-analysis of prospective cohort studies. Int. J. Cardiol..

[30] Hansen L., Dragsted L.O., Olsen A., Christensen J., Tjonneland A., Schmidt E.B., Overvad K. (2010). Fruit and vegetable intake and risk of acute coronary syndrome. Br. J. Nutr..

[31] Larsson S.C., Virtamo J., Wolk A. (2013). Total and specific fruit and vegetable consumption and risk of stroke: A prospective study. Atherosclerosis.

[32] Oude Griep L.M., Verschuren W.M.M., Kromhout D., Ocke M.C., Geleijnse J.M. (2011). Colors of fruit and vegetables and 10-year incidence of stroke. Stroke.

[33] Oude Griep L.M., Verschuren W.M.M., Kromhout D., Ocke M.C., Geleijnse J.M. (2011). Raw and processed fruit and vegetable consumption and 10-year stroke incidence in a population-based cohort study in the Netherlands. Eur. J. Clin. Nutr..

[34] Cassidy A., Rimm E.B., O’Reilly E.J., Logroscino G., Kay C., Chiuve S.E., Rexrode K.M. (2012). Dietary flavonoids and risk of stroke in women. Stroke.

[35] Goetz M.E., Judd S.E., Hartman T.J., McClellan W., Anderson A., Vaccarino V. (2016). Flavanone intake is inversely associated with risk of incident ischemic stroke in the REasons for geographic and racial differences in stroke (REGARDS) study. J. Nutr..

[36] Collins R., Peto R., MacMahon S., Hebert P., Fiebach N.H., Eberlein K.A., Godwin J., Qizilbash N., Taylor J.O., Hennekens C.H. (1990). Blood pressure, stroke, and coronary heart disease. Part 2, Short-term reductions in blood pressure: Overview of randomised drug trials in their epidemiological context. Lancet.

[37] Wang L., Manson J.E., Gaziano J.M., Buring J.E., Sesso H.D. (2012). Fruit and vegetable intake and the risk of hypertension in middle-aged and older women. Am. J. Hypertens..

[38] Tsubota-Utsugi M., Ohkubo T., Kikuya M., Metoki H., Kurimoto A., Suzuki K., Fukushima N., Hara A., Asayama K., Satoh H. (2011). High fruit intake is associated with a lower risk of future hypertension determined by home blood pressure measurement: The Ohasama study. J. Hum. Hypertens..

[39] Borgi L., Muraki I., Satija A., Willett W.C., Rimm E.B., Forman J.P. (2016). Fruit and vegetable consumption and the incidence of hypertension in three prospective cohort studies. Hypertension.

[40] Song H.J., Paek Y.J., Choi M.K., Lee H.J. (2014). Gender differences in the relationship between risk of hypertension and fruit intake. Prev. Med..

[41] Chan H.T., Yiu K.H., Wong C.Y., Li S.W., Tam S., Tse H.F. (2013). Increased dietary fruit intake was associated with lower burden of carotid atherosclerosis in Chinese patients with type 2 diabetes mellitus. Diabetic Med..

[42] Zhu Y., Zhang Y., Ling W., Feng D., Wei X., Yang C., Ma J. (2011). Fruit consumption is associated with lower carotid intima-media thickness and C-reactive protein levels in patients with type 2 diabetes mellitus. J. Am. Diet Assoc..

[43] Cassidy A., Bertoia M., Chiuve S., Flint A., Forman J., Rimm E.B. (2016). Habitual intake of anthocyanins and flavanones and risk of cardiovascular disease in men. Am. J. Clin. Nutr..

[44] Li G.L., Zhu Y.N., Zhang Y., Lang J., Chen Y.M., Ling W.H. (2013). Estimated daily flavonoid and stilbene intake from fruits, vegetables, and nuts and associations with lipid profiles in chinese adults. J. Acad. Nutr. Diet..

[45] Bendinelli B., Masala G., Saieva C., Salvini S., Calonico C., Sacerdote C., Agnoli C., Grioni S., Frasca G., Mattiello A. (2011). Fruit, vegetables, and olive oil and risk of coronary heart disease in Italian women: The EPICOR study. Am. J. Clin. Nutr..

[46] Dauchet L., Montaye M., Ruidavets J., Arveiler D., Kee F., Bingham A., Ferrieres J., Haas B., Evans A., Ducimetiere P. (2010). Association between the frequency of fruit and vegetable consumption and cardiovascular disease in male smokers and non-smokers. Eur. J. Clin. Nutr..

[47] Sharma S., Vik S., Kolonel L.N. (2014). Fruit and vegetable consumption, ethnicity and risk of fatal ischemic heart disease. J. Nutr. Health Aging.

[48] Sangita S., Vik S.A., Pakseresht M., Kolonel L.N. (2013). Adherence to recommendations for fruit and vegetable intake, ethnicity and ischemic heart disease mortality. Nutr. Metab. Cardiovasc. Dis..

[49] Rautiainen S., Levitan E.B., Mittleman M.A., Wolk A. (2015). Fruit and vegetable intake and rate of heart failure: A population-based prospective cohort of women. Eur. J. Heart Fail..

[50] Deanfield J., Donald A., Ferri C., Giannattasio C., Halcox J., Halligan S., Lerman A., Mancia G., Oliver J.J., Pessina A.C. (2005). Endothelial function and dysfunction. Part I: Methodological issues for assessment in the different vascular beds: A statement by the working group on endothelin and endothelial factors of the European Society of Hypertension. J. Hypertens..

[51] Thandapilly S.J., LeMaistre J.L., Louis X.L., Anderson C.M., Netticadan T., Anderson H.D. (2012). Vascular and cardiac effects of grape powder in the spontaneously hypertensive rat. Am. J. Hypertens..

[52] Liang Y., Wang J., Gao H.Q., Wang Q.Z., Zhang J., Qiu J. (2016). Beneficial effects of grape seed proanthocyanidin extract on arterial remodeling in spontaneously hypertensive rats via protecting against oxidative stress. Mol. Med. Rep..

[53] Wang X.H., Huang L.L., Yu T.T., Zhu J.H., Shen B., Zhang Y., Wang H.Z., Gao S. (2013). Effects of oligomeric grape seed proanthocyanidins on heart, aorta, kidney in DOCA-salt mice: Role of oxidative stress. Phytother. Res..

[54] Rodriguez-Rodriguez R., Justo M.L., Claro C.M., Vila E., Parrado J., Herrera M.D., de Sotomayor M.A. (2012). Endothelium-dependent vasodilator and antioxidant properties of a novel enzymatic extract of grape pomace from wine industrial waste. Food Chem..

[55] Felice F., Zambito Y., Di Colo G., D’Onofrio C., Fausto C., Balbarini A., Di Stefano R. (2012). Red grape skin and seeds polyphenols: Evidence of their protective effects on endothelial progenitor cells and improvement of their intestinal absorption. Eur. J. Pharm. Biopharm..

[56] Quintieri A.M., Baldino N., Filice E., Seta L., Vitetti A., Tota B., De Cindio B., Cerra M.C., Angelone T. (2013). Malvidin, a red wine polyphenol, modulates mammalian myocardial and coronary performance and protects the heart against ischemia/reperfusion injury. J. Nutr. Biochem..

[57] Cui X., Liu X., Feng H., Zhao S., Gao H. (2012). Grape seed proanthocyanidin extracts enhance endothelial nitric oxide synthase expression through 5′-AMP activated protein kinase/surtuin 1-krupple like factor 2 pathway and modulate blood pressure in ouabain induced hypertensive rats. Biol. Pharm. Bull..

[58] Goutzourelas N., Stagos D., Housmekeridou A., Karapouliou C., Kerasioti E., Aligiannis N., Skaltsounis A.L., Spandidos D.A., Tsatsakis A.M., Kouretas D. (2015). Grape pomace extract exerts antioxidant effects through an increase in GCS levels and GST activity in muscle and endothelial cells. Int. J. Mol. Med..

[59] Luzak B., Kosiorek A., Syska K., Rozalski M., Bijak M., Podsedek A., Balcerczak E., Watala C., Golanski J. (2014). Does grape seed extract potentiate the inhibition of platelet reactivity in the presence of endothelial cells?. Adv. Med. Sci..

[60] Razavi S.M., Gholamin S., Eskandari A., Mohsenian N., Ghorbanihaghjo A., Delazar A., Rashtchizadeh N., Keshtkar-Jahromi M., Argani H. (2013). Red grape seed extract improves lipid profiles and decreases oxidized low-density lipoprotein in patients with mild hyperlipidemia. J. Med. Food.

[61] Leibowitz A., Faltin Z., Perl A., Eshdat Y., Hagay Y., Peleg E., Grossman E. (2014). Red grape berry-cultured cells reduce blood pressure in rats with metabolic-like syndrome. Eur. J. Nutr..

[62] Caimari A., Del Bas J.M., Crescenti A., Arola L. (2013). Low doses of grape seed procyanidins reduce adiposity and improve the plasma lipid profile in hamsters. Int. J. Obes..

[63] Eren E., Yilmaz N., Aydin O. (2012). High density lipoprotein and it’s dysfunction. Open Biochem. J..

[64] Zagayko A.L., Kravchenko G.B., Krasilnikova O.A., Ogai Y.O. (2013). Grape polyphenols increase the activity of HDL enzymes in old and obese rats. Oxid. Med. Cell. Longev..

[65] Malinowska J., Oleszek W., Stochmal A., Olas B. (2013). The polyphenol-rich extracts from black chokeberry and grape seeds impair changes in the platelet adhesion and aggregation induced by a model of hyperhomocysteinemia. Eur. J. Nutr..

[66] Safwen K., Selima S., Mohamed E., Ferid L., Pascal C., Mohamed A., Ezzedine A., Meherzia M. (2015). Protective effect of grape seed and skin extract on cerebral ischemia in rat: Implication of transition metals. Int. J. Stroke.

[67] Zhao G., Gao H., Qiu J., Lu W., Wei X. (2010). The molecular mechanism of protective effects of grape seed proanthocyanidin extract on reperfusion arrhythmias in rats in vivo. Biol. Pharm. Bull..

[68] Zhang Y., Shi H., Wang W., Ke Z., Xu P., Zhong Z., Li X., Wang S. (2011). Antithrombotic effect of grape seed proanthocyanidins extract in a rat model of deep vein thrombosis. J. Vasc. Surg..

[69] Stroher D.J., Escobar P.J.C., Gullich A.A., Pilar B.C., Coelho R.P., Bruno J.B., Faoro D., Manfredini V. (2015). 14 Days of supplementation with blueberry extract shows anti-atherogenic properties and improves oxidative parameters in hypercholesterolemic rats model. Int. J. Food Sci. Nutr..

[70] Rodriguez-Mateos A., Ishisaka A., Mawatari K., Vidal-Diez A., Spencer J.P., Terao J. (2013). Blueberry intervention improves vascular reactivity and lowers blood pressure in high-fat-, high-cholesterol-fed rats. Br. J. Nutr..

[71] Xie C., Kang J., Chen J.R., Nagarajan S., Badger T.M., Wu X. (2011). Phenolic acids are in vivo atheroprotective compounds appearing in the serum of rats after blueberry consumption. J. Agric. Food Chem..

[72] Cao K., Xu J., Pu W.J., Dong Z.Z., Sun L., Zang W.J., Gao F., Zhang Y., Feng Z.H., Liu J.K. (2015). Punicalagin, an active component in pomegranate, ameliorates cardiac mitochondrial impairment in obese rats via AMPK activation. Sci. Rep..

[73] Al-Jarallah A., Igdoura F., Zhang Y., Tenedero C.B., White E.J., MacDonald M.E., Igdoura S.A., Trigatti B.L. (2013). The effect of pomegranate extract on coronary artery atherosclerosis in SR-BI/APOE double knockout mice. Atherosclerosis.

[74] Sun W.Y., Yan C.H., Frost B., Wang X., Hou C., Zeng M.Q., Gao H.L., Kang Y.M., Liu J.K. (2016). Pomegranate extract decreases oxidative stress and alleviates mitochondrial impairment by activating AMPK-Nrf2 in hypothalamic paraventricular nucleus of spontaneously hypertensive rats. Sci. Rep..

[75] Hajipour S., Sarkaki A., Mohammad S., Mansouri T., Pilevarian A., RafieiRad M. (2014). Motor and cognitive deficits due to permanent cerebral hypoperfusion/ischemia improve by pomegranate seed extract in rats. Pak. J. Biol. Sci..

[76] Serra A.T., Rocha J., Sepodes B., Matias A.A., Feliciano R.P., de Carvalho A., Bronze M.R., Duarte C.M.M., Figueira M.E. (2012). Evaluation of cardiovascular protective effect of different apple varieties—Correlation of response with composition. Food Chem..

[77] Gonzalez J., Donoso W., Sandoval N., Reyes M., Gonzalez P., Gajardo M., Morales E., Neira A., Razmilic I., Yuri J.A. (2015). Apple peel supplemented diet reduces parameters of metabolic syndrome and atherogenic progression in ApoE^−/−^ mice. Evid. Based Complement. Altern..

[78] Hu H.J., Luo X.G., Dong Q.Q., Mu A., Shi G.L., Wang Q.T., Chen X.Y., Zhou H., Zhang T.C., Pan L.W. (2016). Ethanol extract of Zhongtian hawthorn lowers serum cholesterol in mice by inhibiting transcription of 3-hydroxy-3-methylglutaryl-CoA reductase via nuclear factor-kappa B signal pathway. Exp. Biol. Med..

[79] Li T.P., Zhu R.G., Dong Y.P., Liu Y.H., Li S.H., Chen G. (2013). Effects of pectin pentaoligosaccharide from hawthorn (*Crataegus pinnatifida* Bunge. Var. Major) on the activity and mRNA levels of enzymes involved in fatty acid oxidation in the liver of mice fed a high-fat diet. J. Agric. Food Chem..

[80] Zhang Y.Y., Zhang L., Geng Y., Geng Y.H. (2014). Hawthorn fruit attenuates atherosclerosis by improving the hypolipidemic and antioxidant activities in apolipoprotein E-Deficient mice. J. Atheroscler. Thromb..

[81] Zhang J., Liang R., Wang L., Yan R., Hou R., Gao S., Yang B. (2013). Effects of an aqueous extract of *Crataegus pinnatifida* Bge. Var. Major N.E.Br. fruit on experimental atherosclerosis in rats. J. Ethnopharmacol..

[82] Duarte P.F., Chaves M.A., Borges C.D., Mendonca C. (2016). Avocado: Characteristics, health benefits and uses. Cienc. Rural.

[83] Villa-Rodriguez J.A., Molina-Corral F.J., Ayala-Zavala J.F., Olivas G.I., Gonzalez-Aguilar G.A. (2011). Effect of maturity stage on the content of fatty acids and antioxidant activity of ‘Hass’ avocado. Food Res. Int..

[84] Rodriguez-Sanchez D.G., Flores-Garcia M., Silva-Platas C., Rizzo S., Torre-Amione G., De la Pena-Diaz A., Hernandez-Brenes C., Garcia-Rivas G. (2015). Isolation and chemical identification of lipid derivatives from avocado (*Persea americana*) pulp with antiplatelet and antithrombotic activities. Food Funct..

[85] Carvajal-Zarrabal O., Nolasco-Hipolito C., Aguilar-Uscanga M.G., Melo-Santiesteban G., Hayward-Jones P.M., Barradas-Dermitz D.M. (2014). Avocado oil supplementation modifies cardiovascular risk profile markers in a rat model of sucrose-induced metabolic changes. Dis. Markers.

[86] Carvajal-Zarrabal O., Nolasco-Hipolito C., Aguilar-Uscanga M.G., Santiesteban G.M., Hayward-Jones P.M., Barradas-Dermitz D.M. (2014). Effect of dietary intake of avocado oil and olive oil on biochemical markers of liver function in sucrose-fed rats. Biomed. Res. Int..

[87] Dabas D., Shegog R.M., Ziegler G.R., Lambert J.D. (2013). Avocado (*Persea americana*) seed as a source of bioactive phytochemicals. Curr. Pharm. Des..

[88] Czompa A., Gyongyosi A., Czegledi A., Csepanyi E., Bak I., Haines D.D., Tosaki A., Lekli I. (2014). Cardioprotection afforded by sour cherry seed kernel: The role of heme oxygenase-1. J. Cardiovasc. Pharmacol..

[89] Li C., He J., Gao Y., Xing Y., Hou J., Tian J. (2014). Preventive effect of total flavones of *Choerospondias axillaries* on ischemia/reperfusion-induced myocardial infarction-related MAPK signaling pathway. Cardiovasc. Toxicol..

[90] Zapata-Sudo G., Da S.J., Pereira S.L., Souza P.J., de Moura R.S., Sudo R.T. (2014). Oral treatment with *Euterpe oleracea* Mart. (acai) extract improves cardiac dysfunction and exercise intolerance in rats subjected to myocardial infarction. BMC Complement. Altern. Med..

[91] Mauray A., Felgines C., Morand C., Mazur A., Scalbert A., Milenkovic D. (2012). Bilberry anthocyanin-rich extract alters expression of genes related to atherosclerosis development in aorta of apo E-deficient mice. Nutr. Metab. Cardiovasc. Dis..

[92] Ash M.M., Wolford K.A., Carden T.J., Hwang K.T., Carr T.P. (2011). Unrefined and refined black raspberry seed oils significantly lower triglycerides and moderately affect cholesterol metabolism in male Syrian hamsters. J. Med. Food.

[93] Yang F., Suo Y.R., Chen D.L., Tong L. (2016). Protection against vascular endothelial dysfunction by polyphenols in sea buckthorn berries in rats with hyperlipidemia. Biosci. Trends.

[94] Fujiwara Y., Hayashida A., Tsurushima K., Nagai R., Yoshitomi M., Daiguji N., Sakashita N., Takeya M., Tsukamoto S., Ikeda T. (2011). Triterpenoids isolated from *Zizyphus jujuba* inhibit foam cell formation in macrophages. J. Agric. Food Chem..

[95] Ono M., Yasuda S., Komatsu H., Fujiwara Y., Takeya M., Nohara T. (2014). Triterpenoids from the fruits and leaves of the blackberry (*Rubus allegheniensis*) and their inhibitory activities on foam cell formation in human monocyte-derived macrophage. Nat. Prod. Res..

[96] Konta E.M., Almeida M.R., Do A.C., Darin J.D., de Rosso V.V., Mercadante A.Z., Antunes L.M., Bianchi M.L. (2014). Evaluation of the antihypertensive properties of yellow passion fruit pulp (*Passiflora edulis* Sims f. Flavicarpa Deg.) in spontaneously hypertensive rats. Phytother. Res..

[97] Furuuchi R., Sakai H., Hirokawa N., Watanabe Y., Yokoyama T., Hirayama M. (2012). Antihypertensive effect of boysenberry seed polyphenols on spontaneously hypertensive rats and identification of orally absorbable proanthocyanidins with vasorelaxant activity. Biosci. Biotechnol. Biochem..

[98] De Souza M.O., Souza E.S.L., de Brito M.C., de Figueiredo B.B., Costa D.C., Silva M.E., Pedrosa M.L. (2012). The hypocholesterolemic activity of acai (*Euterpe oleracea* Mart.) is mediated by the enhanced expression of the ATP-binding cassette, subfamily G transporters 5 and 8 and low-density lipoprotein receptor genes in the rat. Nutr. Res..

[99] Pujari R.R., Vyawahare N.S., Kagathara V.G. (2011). Evaluation of antioxidant and neuroprotective effect of date palm (*Phoenix dactylifera* L.) against bilateral common carotid artery occlusion in rats. Indian J. Exp. Biol..

[100] Varela C.E., Fromentin E., Roller M., Villarreal F., Ramirez-Sanchez I. (2016). Effects of a natural extract of *Aronia melanocarpa* berry on endothelial cell nitric oxide production. J. Food Biochem..

[101] Zhao R.Z., Le K., Li W.D., Ren S., Moghadasian M.H., Beta T., Shen G.X. (2014). Effects of Saskatoon berry powder on monocyte adhesion to vascular wall of leptin receptor-deficient diabetic mice. J. Nutr. Biochem..

[102] Zhao R.Z., Xie X.P., Le K., Li W.D., Moghadasian M.H., Beta T., Shen G.X. (2015). Endoplasmic reticulum stress in diabetic mouse or glycated LDL-treated endothelial cells: Protective effect of Saskatoon berry powder and cyanidin glycans. J. Nutr. Biochem..

[103] Torres-Urrutia C., Guzman L., Schmeda-Hirschmann G., Moore-Carrasco R., Alarcon M., Astudillo L., Gutierrez M., Carrasco G., Yuri J.A., Aranda E. (2011). Antiplatelet, anticoagulant, and fibrinolytic activity in vitro of extracts from selected fruits and vegetables. Blood Coagul. Fibrinolysis.

[104] Kono R., Okuno Y., Nakamura M., Inada K., Tokuda A., Yamashita M., Hidaka R., Utsunomiya H. (2013). Peach (*Prunus persica*) extract inhibits angiotensin II-induced signal transduction in vascular smooth muscle cells. Food Chem..

[105] Isaak C.K., Petkau J.C., O K., Debnath S.C., Siow Y.L. (2015). Manitoba Lingonberry (*Vaccinium vitis-idaea*) bioactivities in ischemia-reperfusion injury. J. Agric. Food Chem..

[106] Lee S.G., Kim B., Yang Y., Pham T.X., Park Y.K., Manatou J.E., Koo S.I., Chun O.K., Lee J.Y. (2014). Berry anthocyanins suppress the expression and secretion of proinflammatory mediators in macrophages by inhibiting nuclear translocation of NF-kappa B independent of NRF2-mediated mechanism. J. Nutr. Biochem..

[107] Rosenblat M., Volkova N., Borochov-Neori H., Judeinstein S., Aviram M. (2015). Anti-atherogenic properties of date vs. Pomegranate polyphenols: The benefits of the combination. Food Funct..

[108] Li S.H., Zhao P., Tian H.B., Chen L.H., Cui L.Q. (2015). Effect of grape polyphenols on blood pressure: A meta-analysis of randomized controlled trials. PLoS ONE.

[109] Evans M., Wilson D., Guthrie N. (2014). A randomized, double-blind, placebo-controlled, pilot study to evaluate the effect of whole grape extract on antioxidant status and lipid profile. J. Funct. Foods.

[110] Rahbar A.R., Mahmoudabadi M., Islam M.S. (2015). Comparative effects of red and white grapes on oxidative markers and lipidemic parameters in adult hypercholesterolemic humans. Food Funct..

[111] Yubero N., Sanz-Buenhombre M., Guadarrama A., Villanueva S., Carrion J.M., Larrarte E., Moro C. (2013). LDL cholesterol-lowering effects of grape extract used as a dietary supplement on healthy volunteers. Int. J. Food Sci. Nutr..

[112] Li S.H., Tian H.B., Zhao H.J., Chen L.H., Cui L.Q. (2013). The acute effects of grape polyphenols supplementation on endothelial function in adults: Meta-Analyses of controlled trials. PLoS ONE.

[113] Alvarez-Suarez J.M., Giampieri F., Tulipani S., Casoli T., Di Stefano G., Gonzalez-Paramas A.M., Santos-Buelga C., Busco F., Quiles J.L., Cordero M.D. (2014). One-month strawberry-rich anthocyanin supplementation ameliorates cardiovascular risk, oxidative stress markers and platelet activation in humans. J. Nutr. Biochem..

[114] Basu A., Betts N.M., Nguyen A., Newman E.D., Fu D., Lyons T.J. (2014). Freeze-dried strawberries lower serum cholesterol and lipid peroxidation in adults with abdominal adiposity and elevated serum lipids. J. Nutr..

[115] Basu A., Fu D.X., Wilkinson M., Simmons B., Wu M., Betts N.M., Du M., Lyons T.J. (2010). Strawberries decrease atherosclerotic markers in subjects with metabolic syndrome. Nutr. Res..

[116] Ellis C.L., Edirisinghe I., Kappagoda T., Burton-Freeman B. (2011). Attenuation of meal-induced inflammatory and thrombotic responses in overweight men and women after 6-week daily strawberry (Fragaria) intake-a randomized placebo-controlled trial. J. Atheroscler. Thromb..

[117] Burton-Freeman B., Linares A., Hyson D., Kappagoda T. (2010). Strawberry modulates LDL oxidation and postprandial lipemia in response to high-fat meal in overweight hyperlipidemic men and women. J. Am. Coll. Nutr..

[118] Udani J.K., Singh B.B., Singh V.J., Barrett M.L. (2011). Effects of acai (*Euterpe oleracea* Mart.) berry preparation on metabolic parameters in a healthy overweight population: A pilot study. Nutr. J..

[119] Kianbakht S., Abasi B., Dabaghian F.H. (2014). Improved lipid profile in hyperlipidemic patients taking vaccinium arctostaphylos fruit hydroalcoholic extract: A randomized double-blind placebo-controlled clinical trial. Phytother. Res..

[120] Larmo P.S., Kangas A.J., Soininen P., Lehtonen H.M., Suomela J.P., Yang B., Viikari J., Ala-Korpela M., Kallio H.P. (2013). Effects of sea buckthorn and bilberry on serum metabolites differ according to baseline metabolic profiles in overweight women: A randomized crossover trial. Am. J. Clin. Nutr..

[121] Lehtonen H.M., Suomela J.P., Tahvonen R., Yang B., Venojarvi M., Viikari J., Kallio H. (2011). Different berries and berry fractions have various but slightly positive effects on the associated variables of metabolic diseases on overweight and obese women. Eur. J. Clin. Nutr..

[122] Qin Y., Xia M., Ma J., Hao Y.T., Liu J., Mou H., Cao L., Ling W.H. (2009). Anthocyanin supplementation improves serum LDL- and HDL-cholesterol concentrations associated with the inhibition of cholesteryl ester transfer protein in dyslipidemic subjects. Am. J. Clin. Nutr..

[123] Chai S.C., Hooshmand S., Saadat R.L., Payton M.E., Brummel-Smith K., Arjmandi B.H. (2012). Daily apple versus dried plum: Impact on cardiovascular disease risk factors in postmenopausal women. J. Acad. Nutr. Diet..

[124] Tenore G.C., Caruso D., Buonomo G., D’Urso E., D’Avino M., Campiglia P., Marinelli L., Novellino E. (2017). Annurca (*Malus pumila* Miller cv. Annurca) apple as a functional food for the contribution to a healthy balance of plasma cholesterol levels: Results of a randomized clinical trial. J. Sci. Food Agric..

[125] Ravn-Haren G., Dragsted L.O., Buch-Andersen T., Jensen E.N., Jensen R.I., Nemeth-Balogh M., Paulovicsova B., Bergstrom A., Wilcks A., Licht T.R. (2013). Intake of whole apples or clear apple juice has contrasting effects on plasma lipids in healthy volunteers. Eur. J. Nutr..

[126] Bondonno C.P., Yang X., Croft K.D., Considine M.J., Ward N.C., Rich L., Puddey I.B., Swinny E., Mubarak A., Hodgson J.M. (2012). Flavonoid-rich apples and nitrate-rich spinach augment nitric oxide status and improve endothelial function in healthy men and women: A randomized controlled trial. Free Radic. Biol. Med..

[127] Hollands W.J., Hart D.J., Dainty J.R., Hasselwander O., Tiihonen K., Wood R., Kroon P.A. (2013). Bioavailability of epicatechin and effects on nitric oxide metabolites of an apple flavanol-rich extract supplemented beverage compared to a whole apple puree: A randomized, placebo-controlled, crossover trial. Mol. Nutr. Food Res..

[128] Stonehouse W., Gammon C.S., Beck K.L., Conlon C.A., von Hurst P.R., Kruger R. (2013). Kiwifruit: Our daily prescription for health. Can. J. Physiol. Pharm..

[129] Gammon C.S., Kruger R., Conlon C.A., von Hurst P.R., Jones B., Stonehouse W. (2014). Inflammatory status modulates plasma lipid and inflammatory marker responses to kiwifruit consumption in hypercholesterolaemic men. Nutr. Metab. Cardiovas..

[130] Gammon C.S., Kruger R., Brown S.J., Conlon C.A., von Hurst P.R., Stonehouse W. (2014). Daily kiwifruit consumption did not improve blood pressure and markers of cardiovascular function in men with hypercholesterolemia. Nutr. Res..

[131] Chang W.H., Liu J.F. (2009). Effects of kiwifruit consumption on serum lipid profiles and antioxidative status in hyperlipidemic subjects. Int. J. Food Sci. Nutr..

[132] Svendsen M., Tonstad S., Heggen E., Pedersen T.R., Seljeflot I., Bohn S.K., Bastani N.E., Blomhoff R., Holme I.M., Klemsdal T.O. (2015). The effect of kiwifruit consumption on blood pressure in subjects with moderately elevated blood pressure: A randomized, controlled study. Blood Press..

[133] Karlsen A., Svendsen M., Seljeflot I., Laake P., Duttaroy A.K., Drevon C.A., Arnesen H., Tonstad S., Blomhoff R. (2013). Kiwifruit decreases blood pressure and whole-blood platelet aggregation in male smokers. J. Hum. Hypertens..

[134] Peou S., Milliard-Hasting B., Shah S.A. (2016). Impact of avocado-enriched diets on plasma lipoproteins: A meta-analysis. J. Clin. Lipidol..

[135] Jenkins D.J.A., Srichaikul K., Kendall C.W.C., Sievenpiper J.L., Abdulnour S., Mirrahimi A., Meneses C., Nishi S., He X., Lee S. (2011). The relation of low glycaemic index fruit consumption to glycaemic control and risk factors for coronary heart disease in type 2 diabetes. Diabetologia.

[136] Terauchi M., Horiguchi N., Kajiyama A., Akiyoshi M., Owa Y., Kato K., Kubota T. (2014). Effects of grape seed proanthocyanidin extract on menopausal symptoms, body composition, and cardiovascular parameters in middle-aged women: A randomized, double-blind, placebo-controlled pilot study. Menopause.

[137] Ras R.T., Zock P.L., Zebregs Y.E., Johnston N.R., Webb D.J., Draijer R. (2013). Effect of polyphenol-rich grape seed extract on ambulatory blood pressure in subjects with pre- and stage I hypertension. Br. J. Nutr..

[138] Tome-Carneiro J., Gonzalvez M., Larrosa M., Yanez-Gascon M.J., Garcia-Almagro F.J., Ruiz-Ros J.A., Garcia-Conesa M.T., Tomas-Barberan F.A., Espin J.C. (2012). One-year consumption of a grape nutraceutical containing resveratrol improves the inflammatory and fibrinolytic status of patients in primary prevention of cardiovascular disease. Am. J. Cardiol..

[139] Tome-Carneiro J., Gonzalvez M., Larrosa M., Yanez-Gascon M.J., Garcia-Almagro F.J., Ruiz-Ros J.A., Tomas-Barberan F.A., Garcia-Conesa M.T., Espin J.C. (2013). Grape resveratrol increases serum adiponectin and downregulates inflammatory genes in peripheral blood mononuclear cells: A triple-blind, placebo-controlled, one-year clinical trial in patients with stable coronary artery disease. Cardiovasc. Drug Ther..

[140] Basu A., Du M., Leyva M.J., Sanchez K., Betts N.M., Wu M., Aston C.E., Lyons T.J. (2010). Blueberries decrease cardiovascular risk factors in obese men and women with metabolic syndrome. J. Nutr..

[141] Johnson S.A., Figueroa A., Navaei N., Wong A., Kalfon R., Ormsbee L.T., Feresin R.G., Elam M.L., Hooshmand S., Payton M.E. (2015). Daily blueberry consumption improves blood pressure and arterial stiffness in postmenopausal women with pre- and stage 1-hypertension: A randomized, double-blind, placebo-controlled clinical trial. J. Acad. Nutr. Diet..

[142] McAnulty L.S., Collier S.R., Landram M.J., Whittaker D.S., Isaacs S.E., Klemka J.M., Cheek S.L., Arms J.C., McAnulty S.R. (2014). Six weeks daily ingestion of whole blueberry powder increases natural killer cell counts and reduces arterial stiffness in sedentary males and females. Nutr. Res..

[143] Riso P., Klimis-Zacas D., Del B.C., Martini D., Campolo J., Vendrame S., Moller P., Loft S., De Maria R., Porrini M. (2013). Effect of a wild blueberry (*Vaccinium angustifolium*) drink intervention on markers of oxidative stress, inflammation and endothelial function in humans with cardiovascular risk factors. Eur. J. Nutr..

[144] Moazen S., Amani R., Homayouni R.A., Shahbazian H., Ahmadi K., Taha J.M. (2013). Effects of freeze-dried strawberry supplementation on metabolic biomarkers of atherosclerosis in subjects with type 2 diabetes: A randomized double-blind controlled trial. Ann. Nutr. Metab..

[145] Alqurashi R.M., Galante L.A., Rowland I.R., Spencer J., Commane D.M. (2016). Consumption of a flavonoid-rich acai meal is associated with acute improvements in vascular function and a reduction in total oxidative status in healthy overweight men. Am. J. Clin. Nutr..

[146] Aghababaee S.K., Vafa M., Shidfar F., Tahavorgar A., Gohari M., Katebi D., Mohammadi V. (2015). Effects of blackberry (*Morus nigra* L.) consumption on serum concentration of lipoproteins, apo A-I, apo B, and high-sensitivity-C-reactive protein and blood pressure in dyslipidemic patients. J. Res. Med. Sci..

[147] Zhao S., Bomser J., Joseph E.L., DiSilvestro R.A. (2013). Intakes of apples or apple polyphenols decease plasma values for oxidized low-density lipoprotein/β_2_-glycoprotein I complex. J. Funct. Foods.

[148] Soriano-Maldonado A., Hidalgo M., Arteaga P., de Pascual-Teresa S., Nova E. (2014). Effects of regular consumption of vitamin C-rich or polyphenol-rich apple juice on cardiometabolic markers in healthy adults: A randomized crossover trial. Eur. J. Nutr..

[149] Auclair S., Chironi G., Milenkovic D., Hollman P.C.H., Renard C.M.G.C., Megnien J., Gariepy J., Paul J., Simon A., Scalbert A. (2010). The regular consumption of a polyphenol-rich apple does not influence endothelial function: A randomised double-blind trial in hypercholesterolemic adults. Eur. J. Clin. Nutr..

[150] Wang L., Bordi P.L., Fleming J.A., Hill A.M., Kris-Etherton P.M. (2015). Effect of a moderate fat diet with and without avocados on lipoprotein particle number, size and subclasses in overweight and obese adults: A randomized, controlled trial. J. Am. Heart Assoc..

[151] Dow C.A., Going S.B., Chow H., Patil B.S., Thomson C.A. (2012). The effects of daily consumption of grapefruit on body weight, lipids, and blood pressure in healthy, overweight adults. Metabolism.

[152] Figueroa A., Wong A., Hooshmand S., Sanchez-Gonzalez M.A. (2013). Effects of watermelon supplementation on arterial stiffness and wave reflection amplitude in postmenopausal women. Menopause.

